# YK-4-279 effectively antagonizes EWS-FLI1 induced leukemia in a transgenic mouse model

**DOI:** 10.18632/oncotarget.5520

**Published:** 2015-10-08

**Authors:** Tsion Zewdu Minas, Jenny Han, Tahereh Javaheri, Sung-Hyeok Hong, Michaela Schlederer, Yasemin Saygideğer-Kont, Haydar Çelik, Kristina M. Mueller, Idil Temel, Metin Özdemirli, Heinrich Kovar, Hayriye Verda Erkizan, Jeffrey Toretsky, Lukas Kenner, Richard Moriggl, Aykut Üren

**Affiliations:** ^1^ Department of Oncology, Georgetown University Medical Center, Washington, DC, USA; ^2^ Department of Pathology, Georgetown University Medical Center, Washington, DC, USA; ^3^ Ludwig Boltzmann Institute for Cancer Research, Vienna, Austria; ^4^ Clinical Institute of Pathology, Medical University of Vienna, Vienna, Austria; ^5^ Unit of Pathology of Laboratory Animals, University of Veterinary Medicine, Vienna, Austria; ^6^ Medical University of Vienna, Vienna, Austria; ^7^ Children's Cancer Research Institute, St. Anna Kinderkrebsforschung, Vienna, Austria; ^8^ Institute of Animal Breeding and Genetics, University of Veterinary Medicine, Vienna, Austria; ^9^ Department of Pediatrics, Medical University of Vienna, Vienna, Austria

**Keywords:** EWS-FLI1, ETS fusion proteins, YK-4-279, ewing sarcoma, erythoid leukemia

## Abstract

Ewing sarcoma is an aggressive tumor of bone and soft tissue affecting predominantly children and young adults. Tumor-specific chromosomal translocations create EWS-FLI1 and similar aberrant ETS fusion proteins that drive sarcoma development in patients. ETS family fusion proteins and over-expressed ETS proteins are also found in acute myeloid leukemia (AML) and acute lymphoblastic leukemia (ALL) patients. Transgenic expression of EWS-FLI1 in mice promotes high penetrance erythroid leukemia with dense hepatic and splenic infiltrations. We identified a small molecule, YK-4-279, that directly binds to EWS-FLI1 and inhibits its oncogenic activity in Ewing sarcoma cell lines and xenograft mouse models. Herein, we tested *in vivo* therapeutic efficacy and potential side effects of YK-4-279 in the transgenic mouse model with EWS-FLI1 induced leukemia. A two-week course of treatment with YK-4-279 significantly reduced white blood cell count, nucleated erythroblasts in the peripheral blood, splenomegaly, and hepatomegaly of erythroleukemic mice. YK-4-279 inhibited EWS-FLI1 target gene expression in neoplastic cells. Treated animals showed significantly better overall survival compared to control mice that rapidly succumbed to leukemia. YK-4-279 treated mice did not show overt toxicity in liver, spleen, or bone marrow. In conclusion, this *in vivo* study highlights the efficacy of YK-4-279 to treat EWS-FLI1 expressing neoplasms and support its therapeutic potential for patients with Ewing sarcoma and other ETS-driven malignancies.

## INTRODUCTION

Ewing sarcoma is a neoplasm of the bone and soft tissue. It is characterized by a pathognomonic chromosomal translocation involving *EWS* and *FLI1* or related genes that occurs in about 95% of cases [1]. EWS is a member of TET family of RNA binding proteins that is ubiquitously expressed [2] with proposed functions in transcription [3, 4], RNA processing [5, 6], and signal transduction [7–9]. On the other hand, FLI1 is a member of ETS family of transcription factors that is predominantly expressed during embryonic development and exclusively in hematopoietic cells in adult tissues [10]. The translocation leads to juxtaposition of the amino-terminal portion of EWS with the carboxy-terminus of FLI1; the fusion protein differs from wild-type proteins based upon loss of the RNA binding domain of EWS and transactivational domain of FLI1 [11]. EWS-FLI1 fusion protein confers its tumorigenic phenotype through aberrant transcriptional activity [12–15] and splicing [16–20].

EWS-FLI1 can transform NIH-3T3 cells [21], primary bone marrow derived mesenchymal progenitors [22], and osteochondrogenic progenitors [23]. In order to efficiently transform cells, both EWS and FLI1 regions of the chimeric transcript are required, although deletions of certain regions modulate its potency [24]. Anti-sense oligodeoxynucleotide or sequence specific siRNA mediated knockdown of *EWS-FLI1* gene expression is detrimental to Ewing sarcoma cells [25–28]. These findings highlight the significance of EWS-FLI1 for initiating and maintaining tumorigenicity of Ewing sarcoma cells; hence its potential for targeted therapy. In addition, EWS-FLI1 is exclusively present in Ewing sarcoma cells, compared to non-tumor somatic cells, further emphasizing EWS-FLI1 as the ideal therapeutic target for Ewing sarcoma [29].

We identified a small molecule inhibitor, YK-4-279, that directly binds to EWS-FLI1 and inhibits its oncogenic activity in Ewing Sarcoma cell lines and xenograft mouse models [30–32]. YK-4-279 showed a significant differential cytotoxicity to cells that harbor EWS-FLI1 compared to those cells that do not, confirming the specificity of this molecule [30, 31]. YK-4-279 blocks specific protein interactions with EWS-FLI1 including DDX5 and RHA leading to altered mRNA splicing [19] as well as transcription [30, 31]. YK-4-279 can also block growth of other ETS driven cancers. YK-4-279 inhibited ERG and ETV1 derived malignant phenotypes in prostate cancer cells both *in vitro* and *in vivo* [33, 34].

Uncertainty about the exact cell type that gives rise to Ewing sarcoma contributes to the lack of *in vivo* models for the disease, which has hindered preclinical study efforts. Torchia *et al* have generated a double transgenic mouse model (referred hereafter as *E/F; Mx1-cre*) where expression of EWS-FLI1 was targeted to the bone marrow, spleen, and liver. This targeted expression resulted from breeding *Rosa26-loxP-stop-loxP-EWS-FLI1 (E/F)* mice with animals that expressed cre recombinase under the pIpC responsive *Mx1*-promoter (*Mx1-cre*) [35]. Expression of EWS-FLI1 in these tissues induced expansion of primitive erythroid/myeloid progenitors leading to rapid leukemia development that caused severe hepatomegaly, splenomegaly, anemia, and 90% mortality within 28 days [35].

Here, we tested the efficacy of YK-4-279 in disrupting the oncogenic activity of EWS-FLI1 *in vivo* using the above model. YK-4-279 treatment inhibited EWS-FLI1 target gene expression including *Mest, Cpne7, c-Myc, Gata1, Gata2,* and *Car8*. We showed that short-term YK-4-279 treatment led to correction of abnormal hematopoiesis and improved overall survival in EWS-FLI1^+^ leukemic mice without overt side effects. We conclude that direct targeting of aberrant ETS transcription factors with small molecules could be of significant therapeutic value in oncology.

## RESULTS

### EWS-FLI1 expression is induced in *E/F; Mx1-cre* transgenic mouse model

**Figure 1:**
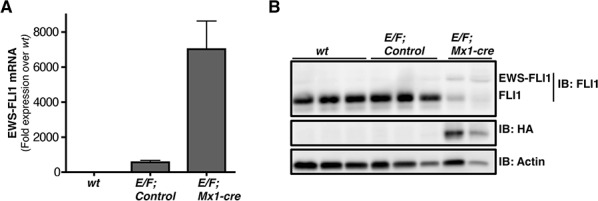
EWS-FLI1 is expressed in E/F; Mx1-cre mouse model **A.** RT-qPCR comparing EWS-FLI1 mRNA level in spleens of *wt* (C57BL/6) (*n* = 3), *E/F; control (*n* = 2)*, vs. *E/F; Mx1-cre* (*n* = 3) mice. **B.** Western blot showing protein levels of EWS-FLI1 in spleens of *E/F; Mx1-cre* transgenic mice compared to *wt* (C57BL/6) mice and *E/F; control* mice that lack *cre* required for EWS-FLI1 activation. Anti-FLI1 antibody epitope is retained in EWS-FLI1. Endogenous FLI1 is highly expressed in the normal mouse spleens. Anti-HA antibody was used to detect the protein level of EWS-FLI1 that is HA tagged. Actin was used as a loading control. Each lane represents an individual animal.

In order to confirm that EWS-FLI1 expression is induced in the *E/F; Mx1-cre* transgenic mouse model, protein and RNA was extracted from spleens of *wt* (C57BL/6) mice, *E/F; control* mice (*E/F* mice that did not inherit *Mx1-cre*), and pIpC induced *E/F; Mx1-cre* mice and were subjected to western blotting and RT-qPCR, respectively. The transgene was detected in the spleens *of E/F; Mx1-cre* mice verifying that the *E/F; Mx1-cre* transgenic mouse model expresses the therapeutic target of interest (Figures 1A and 1B). EWS-FLI1 was not detected at either mRNA or protein level in *wt* mice. EWS-FLI1 mRNA level in *E/F; control* mice was 564 fold over *wt* mice (Figure 1A). This could be due to a leaky read through transcription. However, the expression was not enough to make a detectable protein (Figure 1B). It is possible that the protein level may have been below the detection limit of the technique or the antibody used. Regardless, the leaky expression observed in *E/F; control* mice was not biologically significant since these mice did not develop leukemia over one year follow up.

### CD43^+^, CD71^+^, CD117^+^, CD45^−^, cells are enriched in the spleens and peripheral blood of mice with EWS-FLI1 induced leukemia

**Figure 2:**
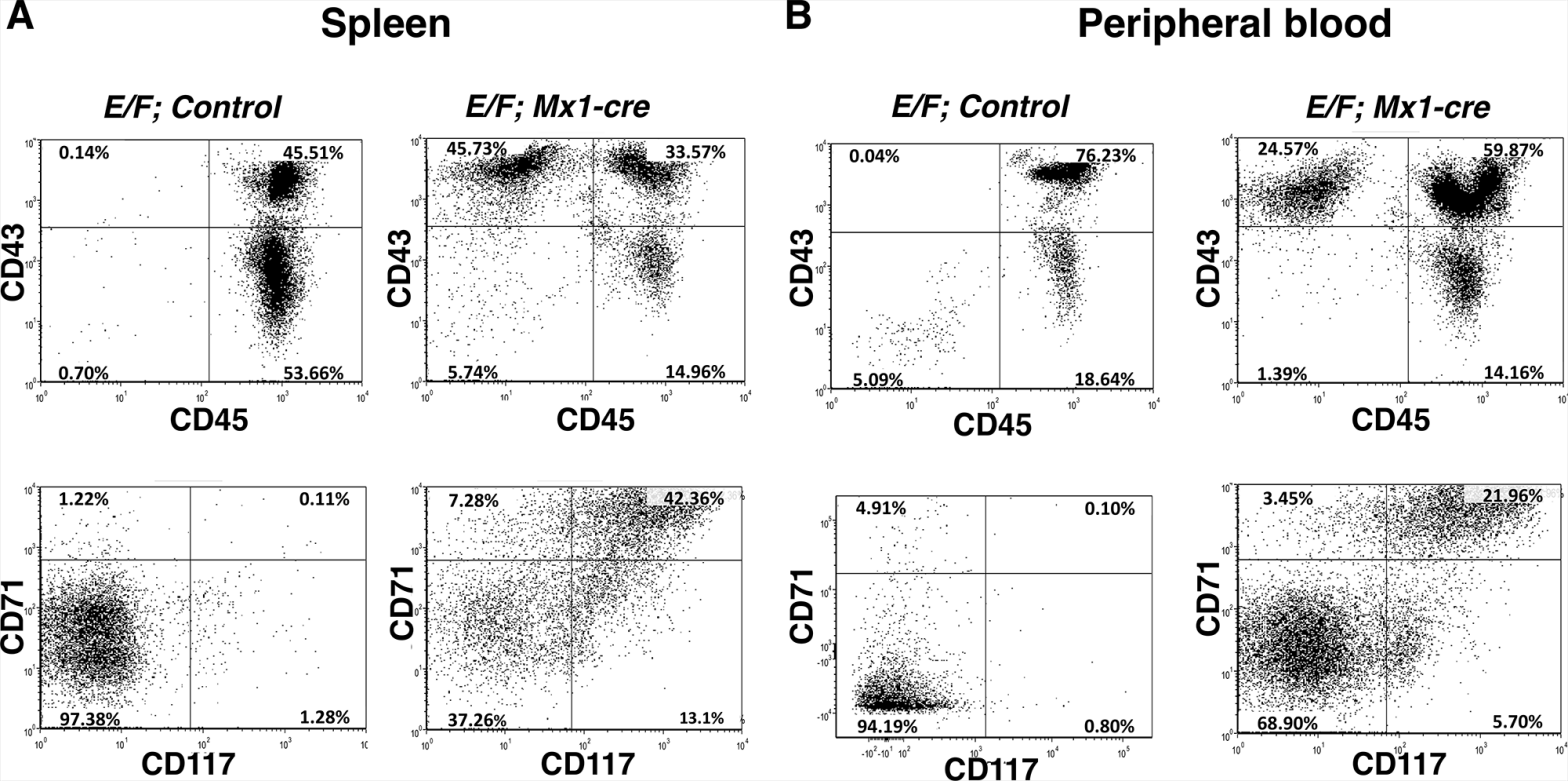
CD43+, CD71+, CD117+, and CD45-, cells are enriched in spleens and peripheral blood of mice with EWS-FLI1 induced leukemia Cells from **A.** spleen and **B.** peripheral circulation of mice with EWS-FLI1-induced leukemia vs. control were evaluated for surface antigen expression. The cells were stained with fluorescently labeled CD43, CD45, CD71, and CD117 antibodies, subsequently analyzed by flow cytometry.

Malignant cells of *E/F; Mx1-cre* mice co-express CD43, CD71, and CD117, but do not express CD45 or CD34 [35]. CD43 (leukosialin) is expressed in early hematopoietic progenitors including those of erythroid lineage that are often CD43^+^, CD34^−^ and CD45^−^ [36]. CD71 (transferrin receptor) is highly expressed in immature erythroid cells with the highest levels in early erythroid precursors through intermediate normoblast phase [37, 38]. CD117 (c-kit) can differentiate erythroblasts from other progenitors when used in combination with other cell surface antigens such as CD45 and CD71. Cells from the spleen and peripheral circulation of leukemic *E/F; Mx1-cre* mice were evaluated for surface antigen expression and showed a phenotype of CD43^+^, CD71^+^, CD117^+^, and CD45^−^. We used this profile to characterize cells from *control* mice and pIpC induced *E/F; Mx1-cre* mice with late stage leukemia. Only 0.14% of the splenocytes from *E/F; control* mice were CD43^+^ and CD45^−^ compared to 45.73% in mice with EWS-FLI1 induced leukemia (Figure 2A). Moreover, only 0.11% of the splenocytes of control mice were CD71^+^ and CD117^+^ compared to 42.36% in mice with EWS-FLI1 induced leukemia (Figure 2A). The enrichment of these populations was also observed in the peripheral blood, where only 0.04% and 0.10% of the cells from peripheral blood of *E/F; control* mice were CD43^+^/CD45^−^ and CD71^+^/CD117^+^, respectively, compared to 24.57% and 21.96% in *E/F; Mx1-cre* mice with leukemia (Figure 2B). Throughout our investigations, we used these biomarkers to monitor leukemia induction and response to treatment.

### EWS-FLI1 induced leukemia display marked increase in clonogenic capacity, which is reduced by YK-4-279

**Figure 3:**
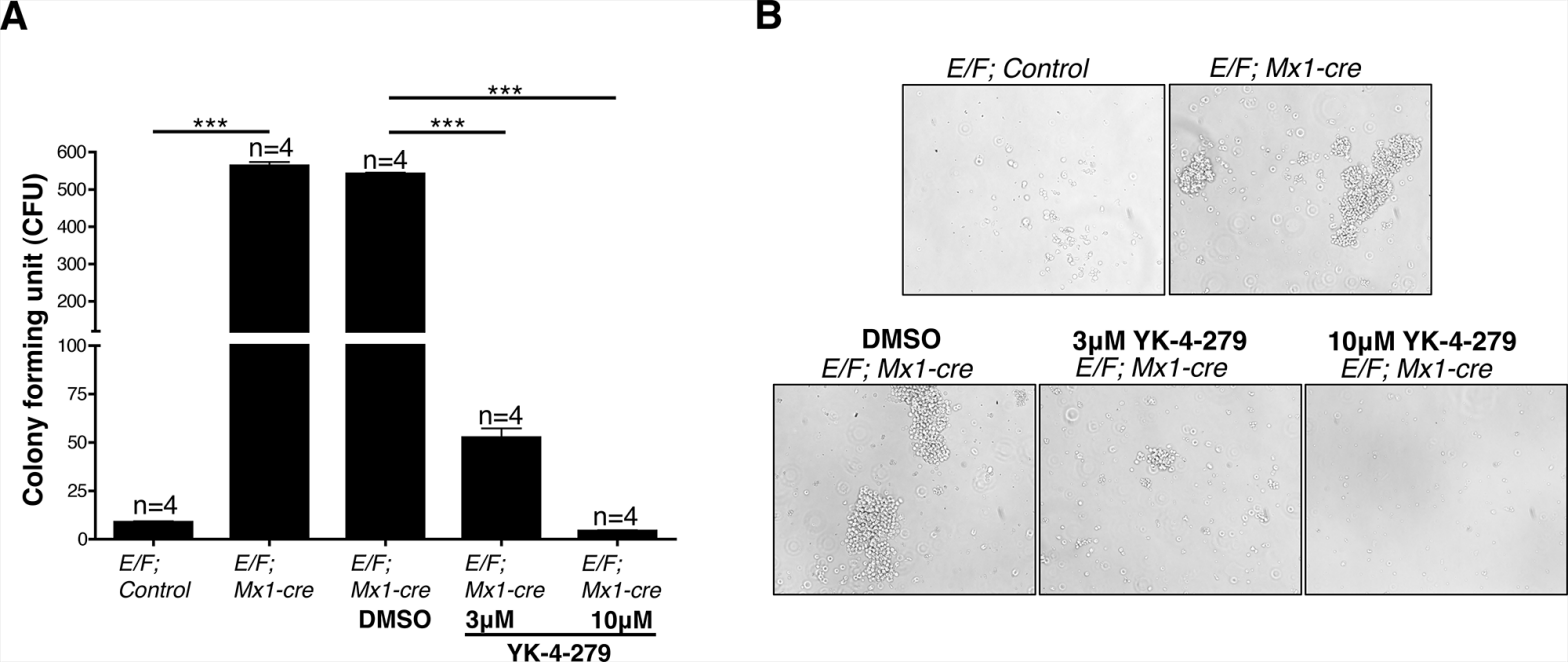
Spleen cells of mice with EWS-FLI1 induced leukemia display significantly increased clonogenic potential, which is inhibited by YK-4-279 **A.** The number of colony forming cells (CFCs) in 1 × 10^5^ spleen cells of leukemic *E/F; Mx1-cre vs. E/F; control* mice was evaluated in an *in vitro* colony forming unit (CFU) assay. Effect of YK-4-279 on colony forming potential of cells from EWS-FLI1 induced leukemic mice was assessed in the presence of vehicle (DMSO) vs. 3 μM or 10 μM YK-4-279. Colonies were counted on the 4^th^ day following plating. ***; *p* < 0.0001. **B.** Representative phase contrast images of CFU colonies from each experimental condition are given.

We analyzed splenocytes for colony forming unit (CFU) in the presence of vehicle (DMSO) compared to treatment with 3 μM or 10 μM YK-4-279 to test the effect of the small molecule on EWS-FLI1 induced leukemia *in vitro*. Splenocytes of healthy adult mice have low numbers of colony forming cells (CFCs). *E/F; control* mouse had an average of 8 CFCs/100,000 cells, while splenocytes of a littermate mouse with EWS-FLI1 induced leukemia of the same sex and age had significantly increased CFCs (an average of 563 CFCs/100,000 cells) suggesting that most of these CFCs are leukemic progenitors (Figure 3A). Treatment of CFC cultures with 3 μM or 10 μM YK-4-279 led to 90% or 99% reduction, respectively, in the clonogenic capacity of leukemic stem cells of the same mouse. In addition, colonies from *E/F; Mx1-cre* mice splenocytes were larger in size than those of control mice (Figure 3B).

### YK-4-279 significantly reduced disease burden *of E/F; Mx1-cre* leukemic mice

**Figure 4:**
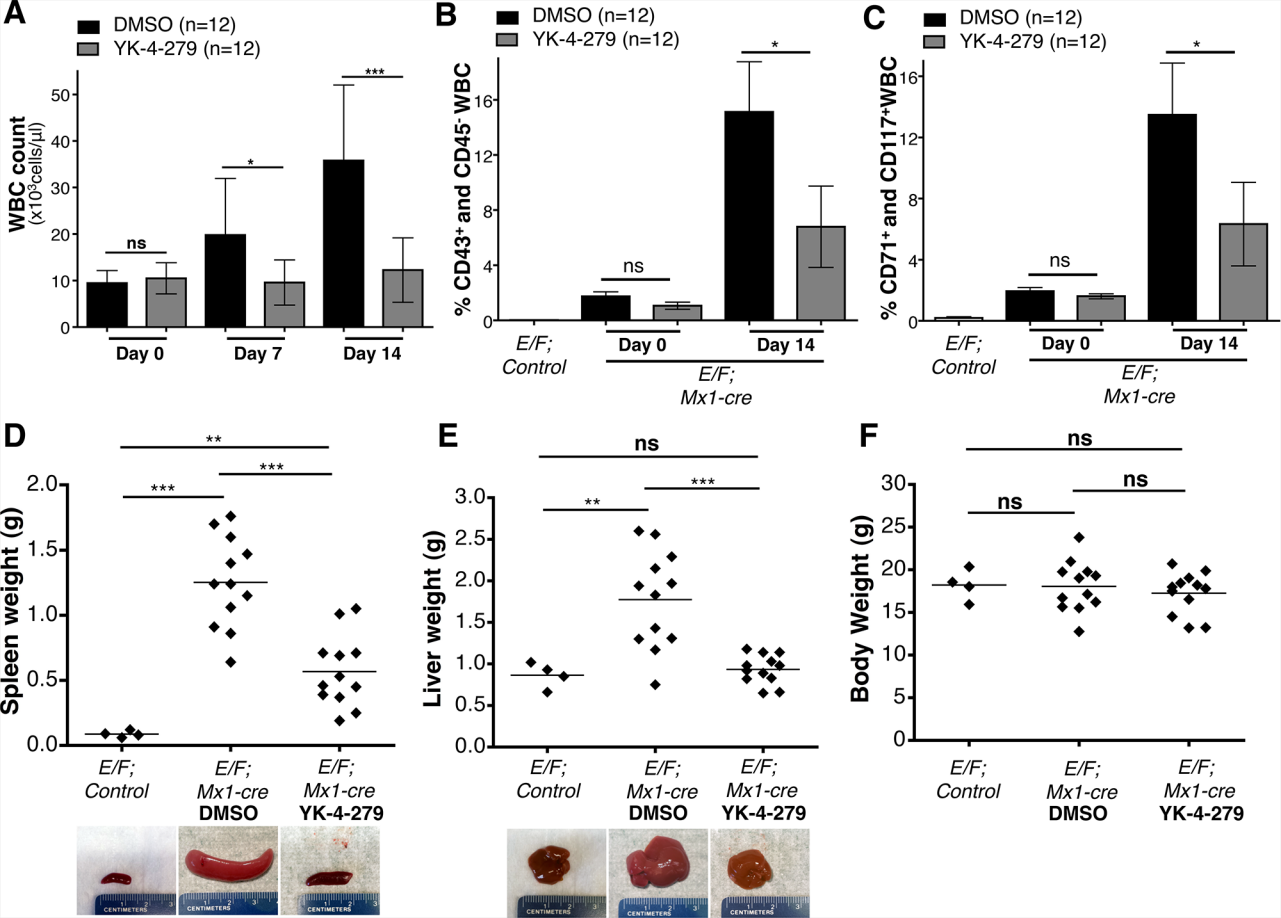
YK-4-279 antagonizes EWS-FLI1 driven erythroleukemia **A.** Effect of YK-4-279 on white blood cell count of leukemic *E/F; Mx1-cre* mice at time of randomization (Day 0), one week after treatment (Day 7), and two weeks following treatment or at the time of euthanasia (Day 14) was assessed. **B.** and **C.** Enrichment of erythroblasts (CD43^+^, CD71^+^, CD117^+^, and CD45^−^ white blood cells) in peripheral circulation at time of randomization to YK-4-279 or DMSO treatment (Day 0) and after two weeks of treatment (Day 14) was evaluated. Erythroblast enrichment in the peripheral blood of pIpC injected *E/F; Mx1-cre* mice was monitored and was compared to healthy *E/F; control* mice to confirm leukemia induction. Mice were assigned to treatment following an average enrichment of CD43^+^/CD45^−^ and CD71^+^/CD117^+^ WBC to 1.41% and 1.75% compared to 0.02% and 0.19% in *E/F; control* mice. The amount of erythroblasts in the peripheral blood after the two weeks long treatment regimen with YK-4-279 vs. DMSO was compared to assess efficacy of YK-4-279 in inhibiting EWS-FLI1 driven erythroleukemia. Additionally post-mortem **D.** spleen, **E.** liver, and **F.** body weights of YK-4-279 or vehicle treated mice were compared to those of healthy *E/F; control* mice of the same age at Day 14 to show the effect of treatment in reducing hepatomegaly and splenomegaly. Representative spleen and liver images of healthy *E/F; control* mice and DMSO or YK-4-279 treated leukemic *E/F; Mx1-cre* mice are given. *; *p* < 0.05, **; *p* < 0.001, ***; *p* < 0.0001, ns; not-significant.

We then investigated the effect of YK-4-279 on disease burden of mice with EWS-FLI1 induced leukemia. *E/F; Mx1-cre* mice were injected with 1 mg pIpC at 1 month of age to activate *Mx1* promoter. The animals were followed up with weekly blood analysis for evidence of disease. Once an increase in white blood cell (WBC) count to an average of 10,000 cells/μl of blood and a 0.4–4% enrichment of CD43^+^, CD71^+^, CD117^+^, and CD45^−^ erythroblasts in the peripheral blood were observed, the mice were randomly assigned to treatment or control groups (Day 0) (Figures 4A–4C). Leukemic *E/F; Mx1-cre* mice were treated with once a day intraperitoneal injections (IP) of YK-4-279 five-times a week for two weeks at a dose of 200 mg/kg for the first two injections followed by 150 mg/kg for the remaining injections. The mice were then euthanized two weeks following the start of therapy (Day14).

**Figure 5:**
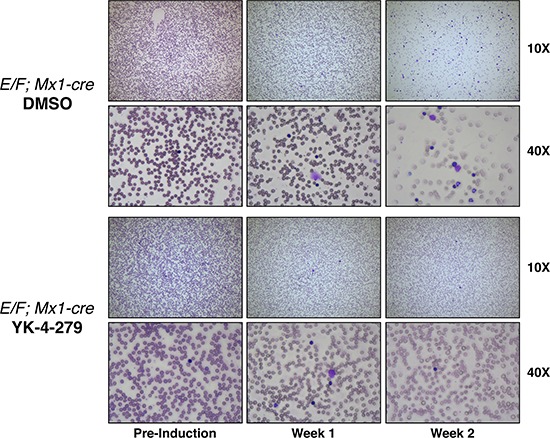
YK-4-279 reduced nucleated erythroblast cells in peripheral blood smears of mice with EWS-FLI1 induced leukemia Representative images of peripheral blood smears of *E/F; Mx1-cre* mice before pIpC induction are given on the left column. Images of blood smears after one week (middle) and two weeks (right) of treatment with YK-4-279 or vehicle are shown. Induction of EWS-FLI1 expression causes a high white blood cell count originating from an erythroblast expansion. This expansion could be efficiently blocked by YK-4-279 treatment.

Weekly WBC counts, RBC counts and blood smears along with post-mortem immunophenotyping and spleen and liver weights were used to assess disease burden. Following one week of treatment, a statistically significant reduction of WBC count occurred in the YK-4-279 treatment group vs. control (*p* < 0.02) (Figure 4A). The treatment effect was even more pronounced after two weeks with significant reduction in WBC count (*p* < 0.0001) (Figure 4A). Overall, YK-4-279 significantly lowered the amount of blast cells in the peripheral blood (Figures 4B and 4C and Figure 5). Following the two weeks treatment, the average enrichment of erythroblasts in the peripheral blood as characterized by CD43^+^/CD45^−^ and CD71^+^/CD117^+^ cells was 15.1% and 13.5% for DMSO group compared to 6.8% and 6.3% for YK-4-279 treatment group (Figure 4B and 4C). EWS-FLI1 induced leukemia is characterized by splenomegaly and hepatomegaly. YK-4-279 significantly reduced the spleen and liver weights of *E/F; Mx1-cre* mice with EWS-FLI1 induced leukemia without affecting the total body weight measured at the time of euthanasia (Figures 4D–4F). Furthermore, YK-4-279 treatment rescued the anemic state of the leukemic mice at late stage (Supplementary Figure S1).

**Figure 6:**
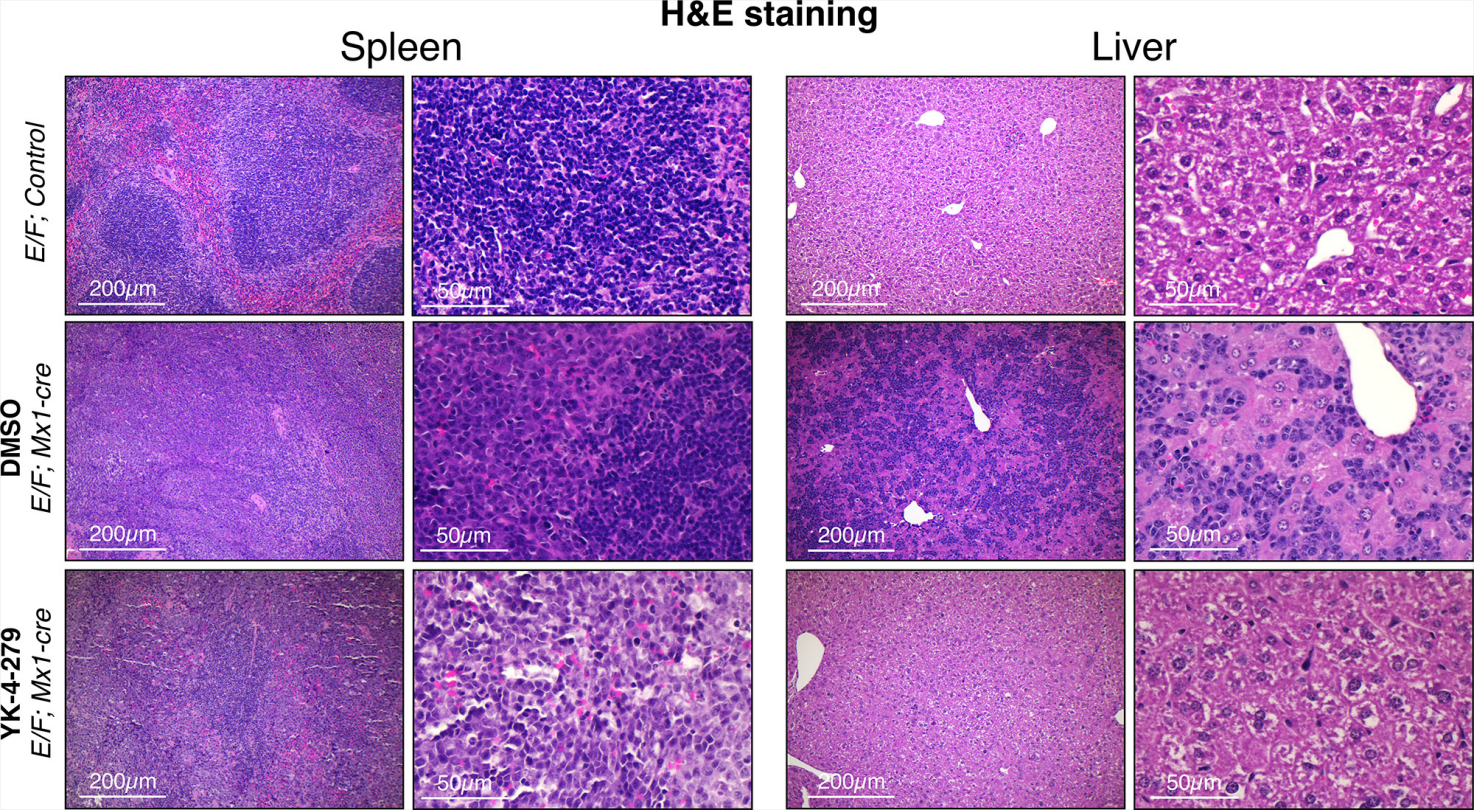
Erythroblast infiltration and expansion was efficiently blocked by YK-4-279 *E/F; Mx1-cre* mice after two weeks treatment with vehicle (middle row) or YK-4-279 (bottom row) were euthanized and spleen and liver samples were processed for histo-pathology analysis. *E/F; control* mice served as healthy controls (top row). *E/F; control* mice do not display any symptoms due to the lack of *Mx1-cre* transgene that allows for cre mediated recombination leading to subsequent *EWS-FLI1* activation. Hematoxylin and Eosin (H&E) staining displays disrupted spleen and liver architecture due to massive infiltration and expansion of erythroblasts. Erythroblast infiltration and expansion was efficiently blocked by two weeks YK-4-279 treatment improving splenic architecture (germinal center formation) and restoring normal liver architecture.

**Figure 7:**
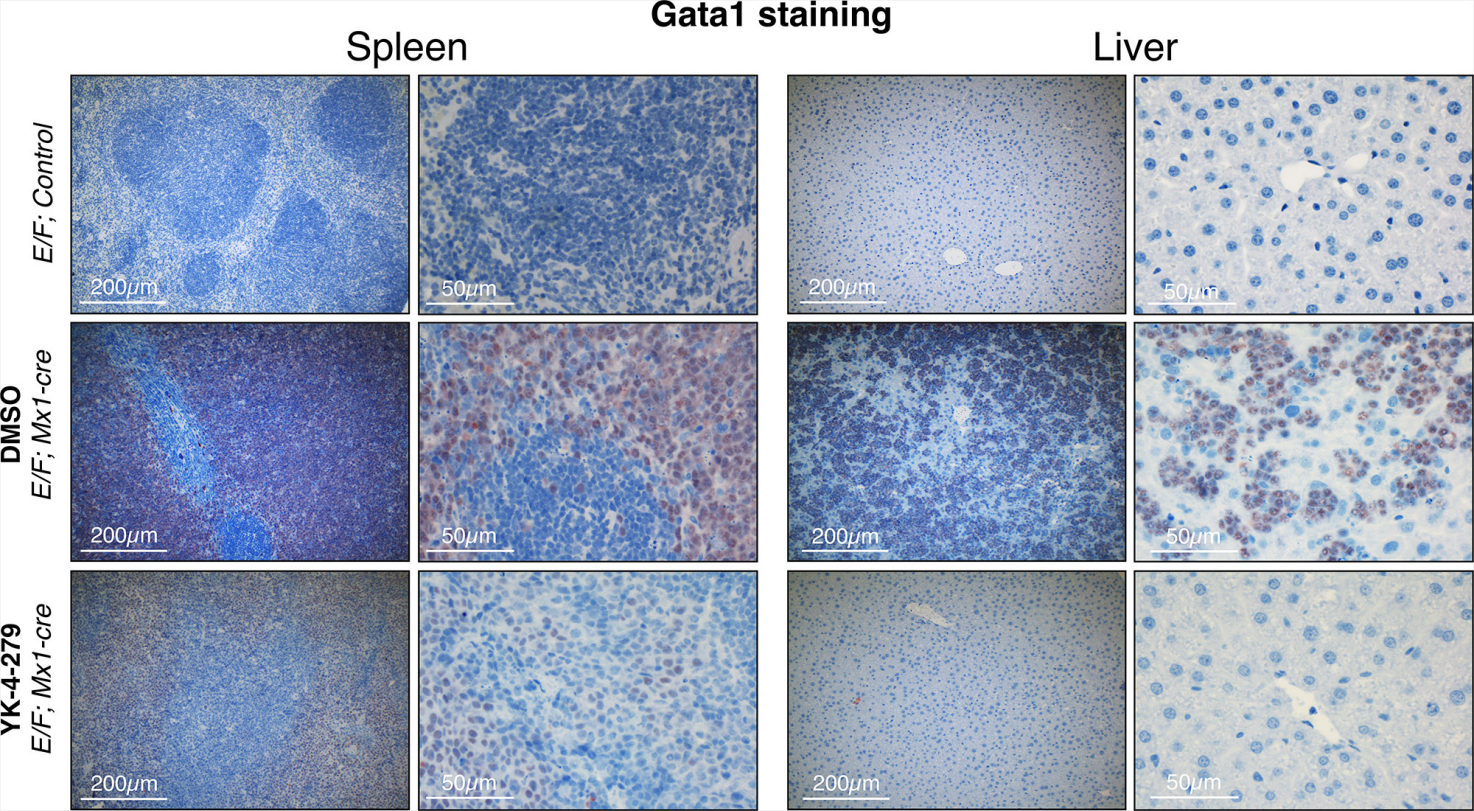
Gata1^+^ erythroblasts were effectively diminished in liver and spleen upon YK-4-279 treatment *E/F; Mx1-cre* mice after two weeks treatment with vehicle (middle row) or YK-4-279 (bottom row) were euthanized and spleen and liver samples were processed for histo-pathology analysis. *E/F; control* mice served as healthy controls (top row) since they lack *cre* required for *EWS-FLI1* activation. EWS-FLI1^+^ erythroblasts stain positive for the erythroid lineage differentiation factor Gata1. A much weaker Gata1 staining in the spleen and liver of YK-4-279 treated mice was observed which is indicative of efficient leukemic disease blockade.

Since *Gata1* is one of the most highly up-regulated genes in erythroblasts of *E/F; Mx1-cre* animals, Gata1 immunostaining was used as a reliable biomarker in the leukemia mouse model to trace erythroblasts. Our findings from IHC staining revealed that Gata1^+^ erythroblasts were effectively diminished in liver and spleen upon YK-4-279 treatment, but DMSO treated mice had dense hepatic infiltration of Gata1^+^ erythroblasts (Figures 6 and 7 and Supplementary Figure S2). Improved spleen and liver architectures were observed upon YK-4-279 treatment in mice compared to severe infiltration of erythroid progenitors in vehicle treated mice.

Evaluation of the bone marrow from *E/F; Mx1-cre* mice showed disease involvement in DMSO treated group, which had reduced number of megakaryocytes, granulocytes and erythroid cells compared to normal mice without the disease (Supplementary Figure S3). We also observed increased number of immature cells and apoptotic cells. When the animals were treated with YK-4-279, the overall morphology of bone marrow showed a significant shift towards normal. Disease burden in bone marrow was also confirmed by immunohistochemistry for Gata1 protein. In normal bone marrow, only the erythroid lineage cells were stained positive for Gata1. EWS-FLI1 induced leukemia resulted in overpopulation of bone marrow with Gata1 positive cells, which was significantly reduced in YK-4-279 treated animals (Supplementary Figure S3). However, a complete elimination of immature cells and restoration of normal structures were not observed, which may explain the disease recurrence following cessation of YK-4-279 treatment after two weeks.

**Figure 8:**
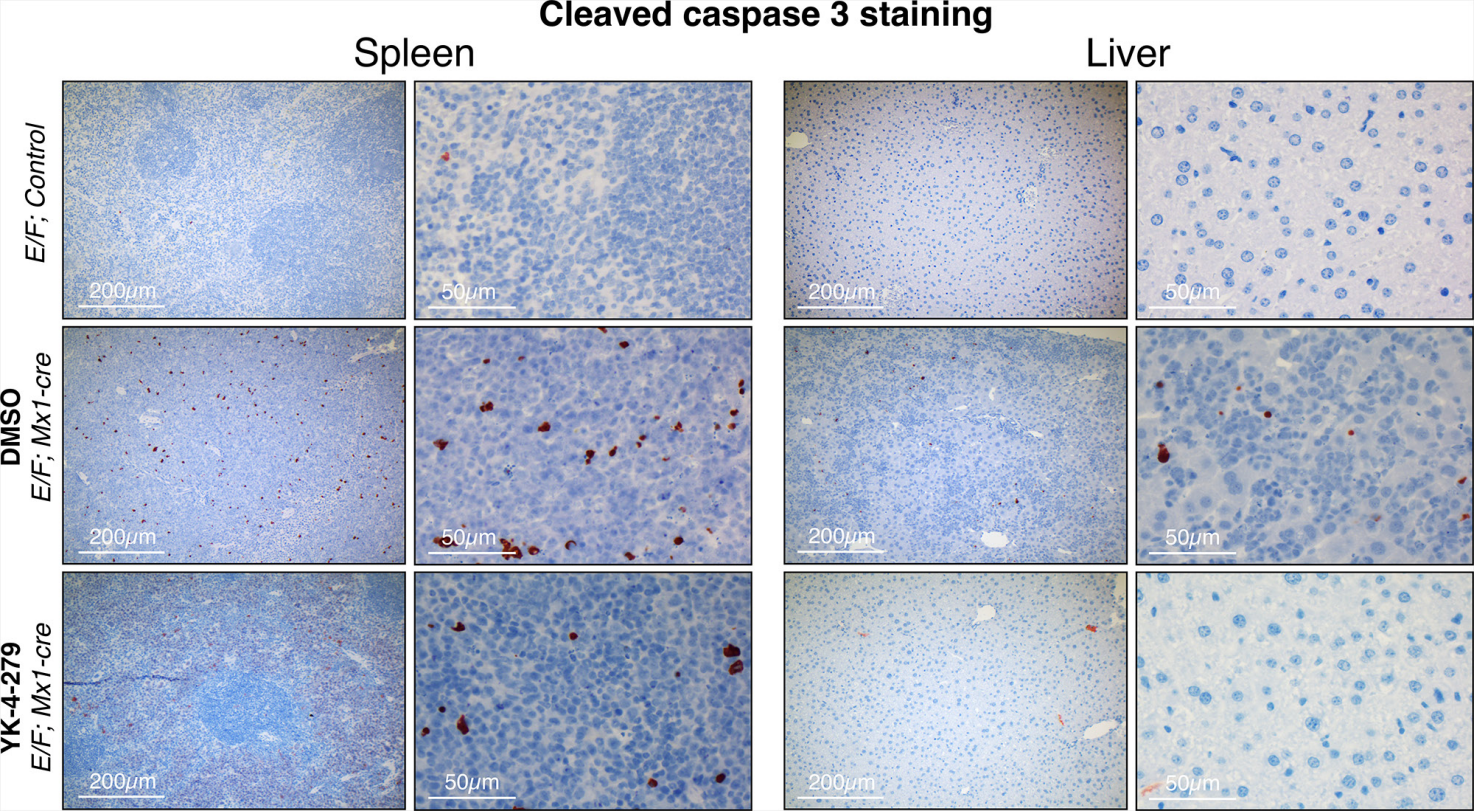
YK-4-279 caused little apoptosis in liver or spleen after two weeks of treatment *E/F; Mx1-cre* mice after two weeks treatment with vehicle (middle row) or YK-4-279 (bottom row) were euthanized and spleen and liver samples were processed for histo-pathology analysis. *E/F; control* mice served as healthy controls (top row) which did not display any symptoms of disease or any peculiar apoptosis or proliferation. Cleaved caspase 3 was utilized as a marker of apoptosis for immunohistochemistry. Improved splenic and liver architectures were noted at the analysis time of two weeks after YK-4-279 treatment. At that time, there were no significant apoptosis detectable in the spleen or liver suggesting a healthy organ.

**Figure 9:**
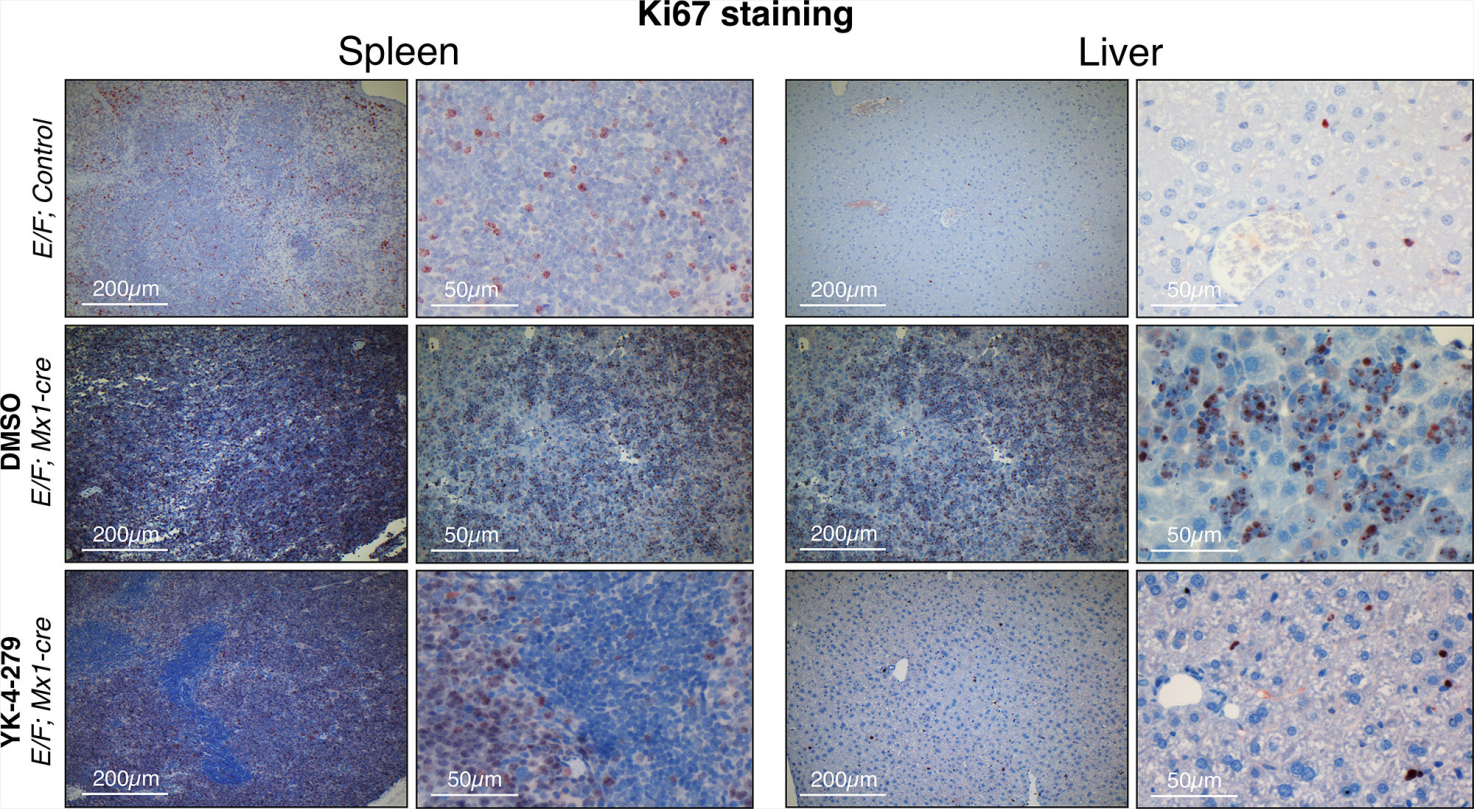
Decreased proliferation was observed in spleens and livers of YK-4-279 treated leukemic mice *E/F; Mx1-cre* mice after two weeks treatment with vehicle (middle row) or YK-4-279 (bottom row) were euthanized and spleen and liver samples were processed for histo-pathology analysis. *E/F; control* mice that lack *cre* required for *EWS-FLI1* activation served as healthy controls (top row) which did not display any symptoms of disease or any peculiar apoptosis or proliferation. Ki67 was used as a marker of proliferation. Ki67 staining of spleen and liver tissues of leukemic *E/F;Mx1-cre* mice treated with YK-4-279 show a decreased proliferation compared to vehicle treated mice.

We measured cleaved caspase 3 as an apoptosis marker and Ki67 as a proliferation marker. *E/F; control* mice without *Mx1-Cre* served as negative controls in immunostainings, which did not display any symptoms of disease or any unusual apoptosis or proliferation. YK-4-279 treatment caused little apoptosis in liver or spleen after two weeks of treatment (Figure 8 and Supplementary Figure S2). The lack of cleaved caspase 3 positive cells in spleens and livers of YK-4-279 treated mice could be due to a number of reasons. Apoptotic erythroblast infiltrates could have been efficiently eradicated by tissue macrophages before the two weeks time point or may have apoptosed prior to infiltrating the spleen or liver. On the other hand, YK-4-279 may have induced other form of cell death that doesn't involve caspase 3. Alternatively, inhibition of EWS-FLI1 function with YK-4-279 may have caused the leukemic cells to differentiate and lose their leukemic property. YK-4-279 may have also induced cell cycle arrest instead of apoptosis, which could not have been recognized by IHC for cleaved caspase 3. We noted a more prominent cleaved caspase 3 staining in splenic infiltration of DMSO treated severely diseased mice (Figure 8 and Supplementary Figure S2). This is indicative of high spontaneous apoptosis in the leukemic cells that appear quite rapidly in the mice with dense splenic infiltrations. The lack of cleaved caspase 3 staining in the normal liver and spleen cells in YK-4-279 treated mice suggests the treatment's minimal toxicity towards the normal cells. This is consistent with the finding that the treatment did not cause liver damage as assessed by serum levels of aspartate aminotransferase (AST) and alanine aminotransferase (ALT) (Supplementary Figure S4). Ki67 staining was very prominent in DMSO treated mice, but YK-4-279 treatment eradicated erythroblasts in the diffuse hepatic infiltrated areas (Figure 9 and Supplementary Figure S2). The difference was less pronounced in the spleen where there was still prominent Ki67 staining in YK-4-279 treated spleens (Figure 9 and Supplementary Figure S2).

### YK-4-279 inhibited EWS-FLI1 regulated genes in *E/F; Mx1-cre* mice

**Figure 10:**
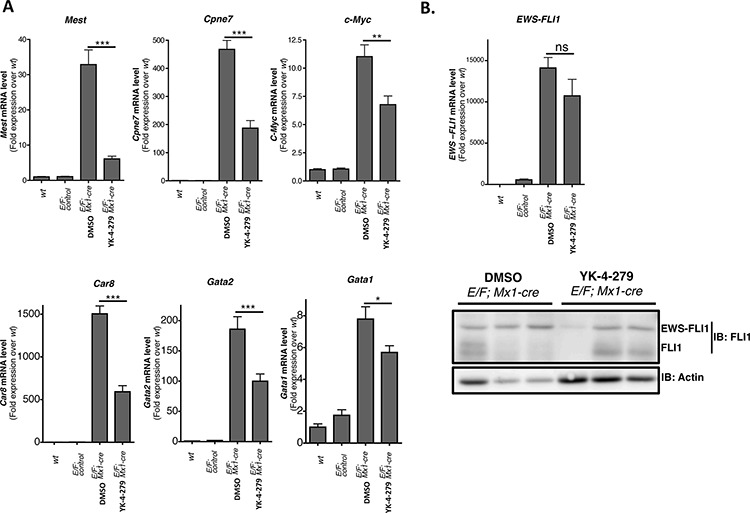
YK-4-279 inhibited genes deregulated by EWS-FLI1 in E/F; Mx1-cre mice **A.** *Mest* (Mesoderm specific transcript), *Cpne7* (Copine VII), *c-Myc, Car8* (Carbonic anhydrase 8), *Gata2* (GATA binding protein 2), and *Gata1* (GATA binding protein 1) are up-regulated when EWS-FLI1 is expressed through *cre* mediated recombination of *Rosa26-loxP-STOP-loxP-EWS-FLI1* site in *E/F; Mx1-cre* mice. The expression of these genes was significantly reduced following two weeks of treatment with YK-4-279 (*n* = 12) over control (*n* = 12). **B.** EWS-FLI1 level did show significant change after two weeks of treatment with YK-4-279 at mRNA and protein level. Each lane in the western blot represents an individual animal. *; *p* < 0.05, **; *p* < 0.001, ***; *p* < 0.0001, ns; not-significant.

One mechanism the fusion gene product promotes Ewing sarcoma is through transcriptional modulation of target genes. We investigated the impact of YK-4-279 on transcriptional activity of EWS-FLI1 in *E/F; Mx1-cre* mice. Surprisingly, previously published EWS-FLI1 target genes were unchanged upon *EWS-FLI1* activation in *E/F; Mx1-cre* mice and the expression profile of EWS-FLI1 induced leukemic cells was different from those of Ewing tumors [35]. EWS-FLI1 may modulate different sets of genes in different cell types to induce leukemia vs. sarcoma. *Mest* (mesoderm specific transcript), *Cpne7* (Copine VII), *c-Myc, Car8* (Carbonic anahydrase VIII), *Gata1* (GATA binding protein 1), and *Gata2* (GATA binding protein 2) are some of the genes differentially regulated in cells with activated EWS-FLI1 in the *E/F; Mx1-cre* mouse model [35]. *Mest, Cpne7, c-Myc, Car8, Gata1,* and *Gata2* were upregulated upon induction of EWS-FLI1 expression in spleens of *E/F; Mx1-cre* mice (Figure 10A). YK-4-279 reversed the levels of these genes close to *E/F; control* mice (Figure 10A). The extent of reduction in *Gata1* expression (Figure 10A) is less marked compared to the level of reduction observed in the IHC data (Figure 7 and Supplementary Figure S2). This could be because of the inherent difference that exists when looking at Gata1 at RNA level in RT-qPCR vs. at protein level in IHC.

The treatment did not significantly affect the levels of EWS-FLI1 at either mRNA or protein (Figure 10B) levels, indicating that the reduction in target gene expression might be due to inhibition of EWS-FLI1 fusion protein's transcriptional activity. The mechanism through which YK-4-279 inhibits the transcriptional activity of EWS-FLI1 is yet to be elucidated. YK-4-279 does not block EWS-FLI1 binding to its cognate DNA binding sites (Supplementary Figure S5). One possible mechanism may entail YK-4-279 disrupting or interfering with proper assembly of the multi-protein complex required for EWS-FLI1 transactivity. RHA is one of the proteins that make up EWS-FLI1 transcriptional complex. Interaction of EWS-FLI1 with RHA regulates its oncogenic properties [39]. Ability of YK-4-279 to block interaction between EWS-FLI1 and RHA [30, 31] or other components of the transcriptional complex may explain YK-4-279's activity against EWS-FLI1 mediated transcription. We observed mitigation in the leukemic phenotype following 2 weeks treatment with YK-4-279 even though EWS-FLI1 level did not significantly alter. Inhibition of EWS-FLI1 function with YK-4-279 may cause the leukemic cells to differentiate and lose their leukemic property. In this case, we may have non-leukemic cells that can still express EWS-FLI1.

### YK-4-279 significantly improves overall survival of *E/F; Mx1-cre* leukemic mice

**Figure 11:**
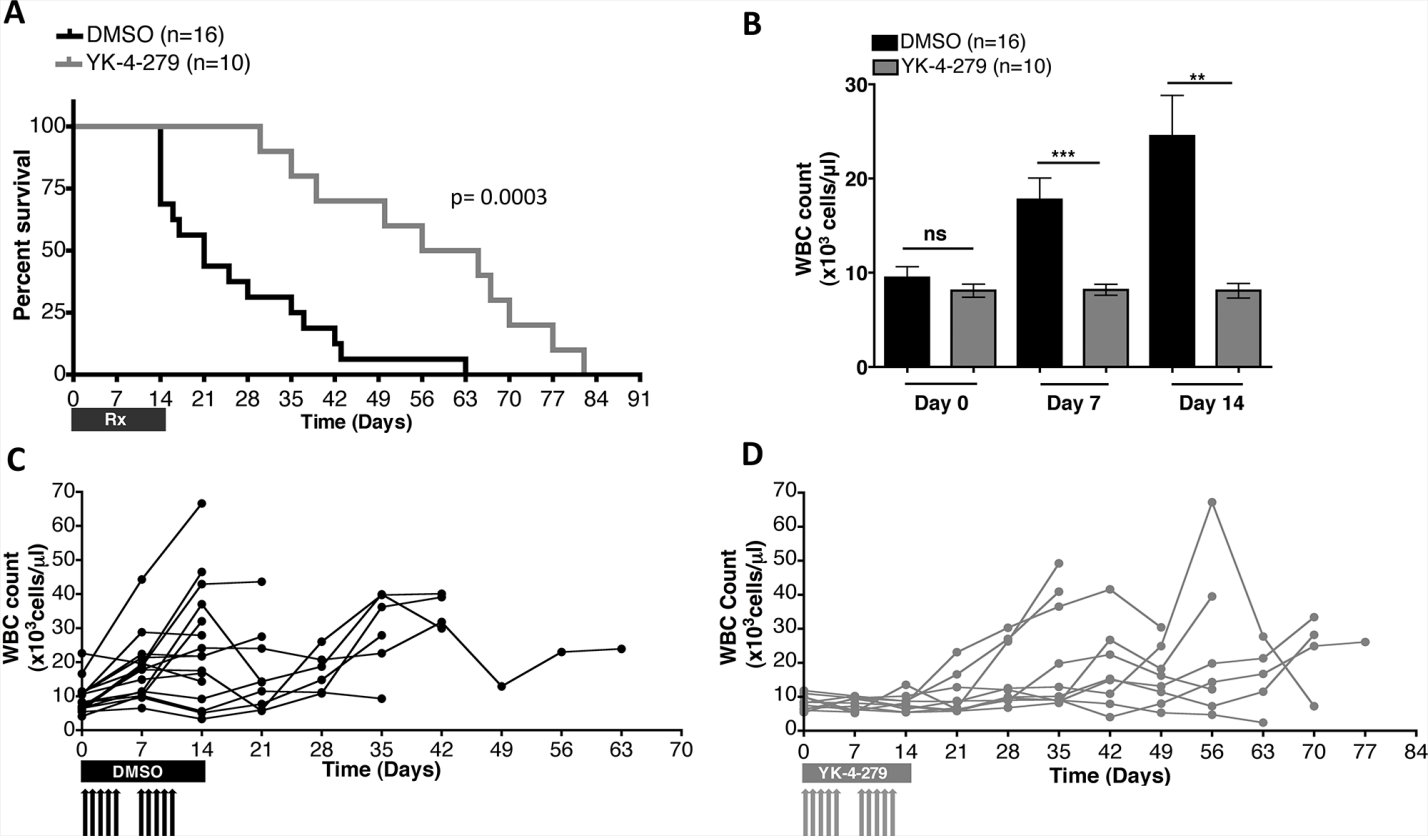
YK-4-279 improved overall survival and disease burden of mice with EWS-FLI1 driven erythroleukemia **A.** Treatment of the transgenic mice daily, five times a week for the first 2 weeks with 150 mg/kg YK-4-279 or vehicle improved overall survival of mice with EWS-FLI1 induced leukemia (*p* = 0.0003). **B.** WBC counts at time of randomization to treatment (Day 0), one week (Day 7), and two weeks (Day 14) following randomization to treatment are given. YK-4-279 treatment regimen led to significant reduction of WBC count. Disease profile of **C.** vehicle vs. **D.** 150 mg/kg YK-4-279 treated individual *E/F; Mx1-cre* mice is presented as weekly WBC counts. **; *p* < 0.001, ***; *p* < 0.0001, ns; not-significant.

To determine if the observed YK-4-279 activity (Figures 3–7) can translate into improved survival of mice with EWS-FLI1 induced leukemia, the animals were allowed to live with erythroleukemia following treatment with daily 150 mg/kg YK-4-279 or vehicle IP injections, five times a week for two weeks in a repeat experiment. A statistically significant improvement in survival of these leukemic mice treated with YK-4-279 was measured where their median survival was 60.5 days as opposed to 21 days for the control group (Figure 11A). During the two weeks of treatment, mice randomized to YK-4-279 had significantly reduced disease burden as monitored by weekly WBC counts. The WBC count of YK-4-279 treated mice compared to controls was lowered by 54% (*p* < 0.0001) and 67% (*p* < 0.002) following one week and two weeks of treatment, respectively (Figure 11B). Follow-up monitoring beyond the two week treatment period showed that most of the mice that received YK-4-279 retained relatively low WBC counts, while those that received vehicle alone had rapid elevation of WBC that led to deterioration of their health (Figure 11C and 11D).

## DISCUSSION

Expression of EWS-FLI1 in cells with the activated *Mx1* promoter in *E/F; Mx1-cre* mice led to leukemia where the mice presented with severe hepatomegaly, splenomegaly, and anemia followed by rapid death [35]. Even though these mice do not develop Ewing sarcoma, the malignancy is driven by EWS-FLI1 making the model relevant to test inhibitors that target EWS-FLI1. Thus, this pre-clinical model was employed to evaluate the small molecule inhibitor YK-4-279 against an EWS-FLI1 driven neoplasm. We show that YK-4-279 significantly reduced clonogenic capacity of leukemic stem cells of *E/F; Mx1-cre* mice *in vitro*. Anti-leukemic effects of YK-4-279 observed in an *in vitro* CFU assay also translated *in vivo*. The treatment led to marked reduction in the spleen and liver weights, WBC count, nucleated erythroblasts in the peripheral blood of mice with EWS-FLI1 induced leukemia. Moreover, the normal spleen and liver architecture that was severely affected in terminally diseased leukemic mice was partially restored upon YK-4-279 treatment. The treatment also improved their anemic state. Furthermore, YK-4-279 treatment extended the survival of *E/F; Mx1-cre* mice with leukemia. These results support the therapeutic potential of YK-4-279 in antagonizing malignant phenotype of EWS-FLI1.

ETS family of transcription factors share a conserved DNA binding domain (ETS domain) all recognizing a purine rich consensus sequence with a central GGAA/T motif [40]. Most ETS transcription factors also harbor a pointed domain (PNT domain) that is important for protein-protein interactions [41]. *ETS* genes have been implicated in a wide array of malignancies including acute myeloid leukemia (AML), acute lymphoblastic leukemia (ALL), chronic myelomonocytic leukemia (CMML), myxoid liposarcoma, Ewing sarcoma, breast cancer, and prostate cancer [42–50]. A subgroup of AML [43, 44, 51] and ALL [42] patients harbor a fusion protein, TLS-ERG, which is similar to EWS-FLI1. EWS and TLS (FUS) belong to the TET family of RNA binding proteins, while FLI1 and ERG belong to the highly conserved ETS family of transcription factors. TLS-ERG [52, 53], EWS-ERG [54], and EWS-FLI1 [1] have all been reported in Ewing sarcoma cases. Additionally, the majority of prostate cancer cases display clear ETS-driver dependence. More than 50% of prostate cancer patients contain chromosomal translocations that involve members of the ETS transcription factor family. The two ETS factors ERG and ETV1 reprogram the androgen receptor cistrome with consequences for androgen ablation therapy in prostate cancer [55, 56]. Moreover, germline or somatic mutations in the *TERT* gene promoter create novel ETS binding sites leading to 2–4 fold increase in TERT protein expression, facilitating immortalization and the override of senescence [57–59]. *TERT* promoter mutations are frequently observed in malignant melanoma, glioblastomas, hepatocellular and bladder carcinomas [60]. Therefore, ETS factors have emerged as candidates for targeted therapy in multiple types of cancers.

YK-4-279 shows therapeutic potential for ETS-driven diseases. Though we still do not know the exact interaction surface, YK-4-279 directly binds to and shows inhibitory activity against EWS-FLI1, ERG, and ETV1 oncoproteins, presumably due to interference with protein-protein interactions [31, 33, 34]. Our group has shown that YK-4-279 binds to ERG and ETV1 and inhibits their activity in prostate cancer cell lines [33] and show anti-metastatic activity *in vivo* [34]. Hence, potential clinical indications of YK-4-279 or similar compounds targeting ETS transcription factors may be significantly expanded.

It is plausible that the ability of YK-4-279 to antagonize the activity of a number of ETS family members could pose a risk of toxicity against tissues that expresses ETS transcription factors as part of their normal physiology. *ETS* genes are highly expressed during embryogenesis and hematopoiesis, while their expression is suppressed in most adult epithelial tissues [61–65]. In this study, YK-4-279 treatment did not suppress but rather enhanced red blood cell counts of *E/F; Mx1-cre* mice compared to those leukemic mice treated with vehicle alone. YK-4-279 caused little apoptosis in normal liver and spleen cells and did not affect liver function as indicated by normal serum levels of liver enzymes. Moreover, YK-4-279 treatment did not lead to body weight loss. Our findings combined with earlier *in vivo* studies [31, 32, 34] suggest that the inhibitor has a reasonable therapeutic window with no overt cytotoxic issues aside from peritoneal thickening observed at the site of intraperitoneal administration. Further analysis of YK-4-279's effect on hematopoietic progenitors cells, WBC differential count, and hemoglobin level may help us better evaluate the potential toxicity of the treatment regiment to normal hematopoiesis. Moreover, detailed toxicology studies in large animals would need to be carried out to assess YK-4-279's safety to humans.

Poor solubility of YK-4-279 limits its dosage, frequency, and duration of murine treatment. Therefore, the mice only received a total of 10 injections. Though the disease was suppressed during the time period that the mice were on YK-4-279, some showed leukemic progression once the treatment was halted (Figure 11D). Nevertheless, two weeks of treatment was enough to result in prolonged survival. Future experiments could utilize a treatment plan with a week-on-week-off schedule or a longer treatment regimen with a lower dose to overcome this problem. Furthermore, better drug formulation may significantly improve the pharmacokinetic properties of YK-4-279. Improved drug formulation may also permit oral administration.

In conclusion, YK-4-279 antagonizes EWS-FLI1 induced leukemia in a transgenic mouse model. This supports the continued preclinical development of the compound for not only Ewing sarcoma patients, but also for a subgroup of AML, ALL and prostate cancer patients with highly homologous translocation products or with a clear ETS-driven gene signature. Future work to further resolve the mechanism of YK-4-279 inhibition will also enhance fusion protein transcription factor targeted therapeutics.

## MATERIALS AND METHODS

All chemicals and reagents were purchased from Sigma-Aldrich unless otherwise specified.

### Immunoblotting

Total protein extracts were prepared from spleens of leukemic or control mice using the following protocol. Small fragments (~2 mm^3^) of flash frozen spleens were homogenized using pestle and motor mixer (VWR, Cat. No: 47747-370) in 200 μl phospholysis buffer (50 mM HEPES pH 7.9, 100 mM sodium chloride, 4.0 mM sodium pyrophosphate, 10 mM EDTA, 10 mM sodium fluoride, and 1% Triton X-100 v:v) containing 2.0 mM sodium vanadate, 1.0 mM PMSF, 4.0 μg/ml aprotinin, and 4.0 μg/ml leupeptin. Once the spleens were fully homogenized, the lysates were incubated on ice for 30 min. Next, the lysates were subjected to centrifugation for 10 min at 16,000 g at 4°C. The supernatants were collected and the protein concentration was determined using Pierce BCA protein assay kit per manufacturer's protocol (Thermoscientific, Cat# 23225). Proteins were denatured in 5x Laemmli sample buffer and subjected to SDS-PAGE (10% polyacrylamide). Resolved proteins were transferred to 0.45 μm Immobilon-P PVDF membrane (Millipore, Cat No. IPVH00010). The membranes were blocked in 5% nonfat dry milk in 1x TTBS (20 mM Tris-HCl, pH 7.5, 150 mM NaCl, 0.5% Tween 20 v:v) for 2 hrs. Dilutions for primary antibodies were anti-FLI1 (Santa Cruz Biotechnology, Cat No. sc-356) at 1:1000, anti-HA (Roche, Cat No. 1867423) at 1:500, and anti-actin-horseradish peroxidase (C-11, Santa Cruz Biotechnology, Cat No. sc-1615) at 1:5000. Primary antibodies were added to the membranes in 5% nonfat dry milk in 1x TTBS for 2 hrs at room temperature. The membranes were then washed three times in 1x TTBS and incubated for 1 h with 1:5000 dilution of horseradish peroxidase-linked anti-rabbit (GE Healthcare, Cat No. LNA934V/AG) or anti-rat secondary antibody (R&D, Cat No. HAF005) prepared in 5% nonfat dry milk. Blots were washed three times in 1x TTBS and then developed using Immobilon Western Chemiluminescent HRP Substrate per the manufacturer's instructions (Millipore Corporation, Cat No. WBKLS0100). Chemiluminescence was detected using a Fujifilm LAS-3000 imaging system.

### RT-qPCR

RNA was extracted from ~50 mg flash frozen spleen fragments using TRIzol according to the manufacturer's protocol (Invitrogen, Cat No. 15596-018). Concentration and purity of RNA was determined using NanoDrop 2000c spectrophotometer (Thermoscientific). Extracted RNA was reverse transcribed to cDNA using QuantiTect reverse transcription kit (Qiagen, Cat No. 205311) as described by the manufacturer using Applied Biosytems Veriti Thermal Cycler. Real-time quantitative PCR was performed in an Eppendorf Mastercycler realplex using KiCqStart SYBR Green qPCR ReadyMix (Sigma-Aldrich, Cat No. KCQS00) per manufacturer's protocol. Data were analyzed for expression relative to 18S rRNA using the comparative Ct method. Forward and reverse qPCR primers used for the study are listed in Supplementary Table S1.

### Chromatin immunoprecipitation (ChIP)

TC71 cells were treated with 10 μM YK-4-279 or DMSO for 2 hours. ChIP was performed using Magna ChIP-A (Millipore, Cat No. 17-610) according to manufacturer's instructions. Briefly, TC71 cells were cross-linked with 1% formaldehyde at room temperature for 10 min. The reaction was stopped with the addition of glycine for 5 min. Nuclear fraction was obtained after cells were lysed. The re-suspended nuclear lysate was sonicated five times for 20 seconds with 30 seconds break on ice. Crosslinked chromatin prepared was subjected to ChIP using 5 μg FLI1 antibody (Santa Cruz C-19, Cat No. sc-356), blocked FLI1 antibody (blocked with 7 times excess blocking peptide (Cat No. sc-356-P)), or IgG- rabbit antibody (Millipore, Cat No. PP64B) in 500 μl volume, overnight at 4°C. Immunoprecipitated DNA was eluted from column and analyzed by qPCR for NR0B1 (5′-GATTCTGTATCAGCTGGTATATACC-3′and 5′-GCATCAGGAAGCCTGGATCC-3′). qPCR was performed in an Eppendorf Mastercycler realplex using KiCqStart SYBR green qPCR ready mix (Sigma, Cat No. KCQS00).

### Surface plasmon resonance (SPR)

Direct binding study between EWS-FLI1 and wildtype vs. mutant oligonucleotide was performed on a Biacore T-200 instrument in the presence of 10 μM YK-4-279 or vehicle. EWS-FLI1 was immobilized onto a CM5 sensor chip (GE Healthcare Bio-Sciences, Piscataway, NJ) using standard amine coupling chemistry in 1X HBS-P running buffer and acetate pH4.0 buffer. The flow rate of ligand immobilization was maintained at 10 μL/min. EWS-FL1 binding to wild-type (ATGTAGACC**GGAA**GTAACTA) and mutant (ATGTAGACC **GCTA**GTAACTA) ETS oligonucleotides. Binding of 100 nM Oligo-WT, 100 nM Oligo-Mutant, 10 μM YK-4-279, and 10 μM YK-4-279 + 100 nM Oligo-WT was evaluated in a running buffer containing 1X HBS-N, 0.05% NDBS, 5% DMSO. The sample flow rate was maintained at 50 μL/min. EWS-FLI1 binding to Oligo-WT in the presence of 10 μM YK-4-279 or vehicle was evaluated using 1:1 kinetic fitting. All the experiments were performed at 25°C.

### Flow cytometry

For cell surface analysis of splenocytes, harvested spleens were minced into small pieces in 1x PBS to release the blood cells, which were subsequently strained using 70 μm cell strainer (Fisher, Cat No. 352350) to prepare a single cell suspension. The strained mix was then subjected to centrifugation at 350 g for 5 min. Pelleted cells were resuspended in 1x PBS and spleen cell count was determined using hemocytometer. 0.25 μl of CD43-PE (Clone S11, BioLegend, Cat No. 143205), 0.5 μl of CD45-Alexa Fluor-488 (Clone 30-F111, BioLegend, Cat No. 103122), 1 μl of CD71-Brilliant Violet 421 (Clone RI7217, BioLegend, Cat No. 113813), and 2.5 μl of CD117-APC (Clone 2B8, BioLegend, Cat No. 105812) fluorescent conjugated primary antibodies were used to stain one million splenocytes in 100 μl 1x PBS for 20 min on ice in the dark. The stained cells were washed twice with 1x PBS. The cells were then resuspended in 500 μl of 1x PBS and analyzed using flow cytometer. For immunofluorescent staining of whole blood, the same amount of fluorochrome conjugated CD43, CD45, CD71, and CD117 primary antibodies were added to 50 μl of EDTA anti-coagulated whole blood. The primary antibodies allowed to incubate for 20 min at room temperature. Red blood cells were then lysed using 2 ml of warm 1x ammonium-chloride-potassium (ACK) lysis buffer (Life Tech., Cat # A10492). The blood-ACK mix was incubated for 10 min at room temperature. Lysed red blood cells were discarded by removing the supernatant following centrifugation at 350 g for 5 min. The stained cells were washed twice with 1x PBS and resuspended to a final volume of 500 μl using 1x PBS and analyzed by FACS.

### Colony forming unit (CFU) assay

Splenocytes were extracted from spleens of *E/F; Control* mice or *E/F; Mx1-cre* mice with activated EWS-FLI1 under sterile conditions. Spleens were minced into small pieces using sterile blade (BD 371222) and were further desegregated by pipetting up and down in Iscove's Modified Dulbecco's Medium + 25 mM HEPES (Stem Cell, Cat No. 36150) to release the blood cells and were subsequently strained using 70 μm cell strainer (Fisher, Cat No. 352350) to prepare a single cell suspension. The strained mix was subjected to centrifugation at 350 g for 5 min at 4°C and the supernatant was discarded. Red blood cells were removed by resuspending the cell pellet in 5 ml ACK lysis buffer (Life Tech., Cat # A10492) followed by incubation at room temperature for 10 min. The cells were pelleted by centrifugation at 350 g for 5 min at 4°C and washed twice with Iscove's MDM containing 2% FBS. Trypan blue exclusion was employed to determine viable cell counts using hemocytometer. Viable splenocytes were prepared in Iscove's MDM with 2% FBS at a concentration of 1 × 10^6^ cells /ml and 0.6 ml (6 × 10^5^ cells) of this 10x cell concentration was added in 6 ml of MethoCult GF M3434 media (StemCell Tech., Cat# 03434). Cells were evenly mixed in the methocult media by briefly vortexing the tube. MethoCult media (1.1 ml) containing 1 × 10^5^ viable splenocytes was dispensed using 16 gauge blunt-end needle (Stem Cell Tech., Cat No. 28110) onto four replicate 35 mm culture dish plates (StemCell Tech., Cat # 27100) per manufacturer's instructions. For CFU assays carried out in the presence of DMSO vs. 3 μM or 10 μM of YK-4-279, the concentrations were first prepared in 100 μl of 2% FBS containing IMDM media and were then vortexed well with 6 ml of methocult media prior to addition of cells. 6 × 10^5^ cells prepared in 500 μl of 2% FBS containing IMDM media were then added to the respective tubes. The cells were mixed with the methocult media containing DMSO or 3 μM YK-4-279 or 10 μM of YK-4-279. MethoCult-drug mixture (1.1 ml) containing 1 × 10^5^ viable splenocytes was dispensed onto 35 mm culture dish plates using 16 gauge blunt-end needle. Number of colonies in the four replicate 35 mm dishes were counted using 60 mm gridded scoring dish (StemCell Tech, Cat# 27500) 4 days after plating.

### *In vivo* experiments

All animal studies were approved by the Georgetown University Institutional Animal Care and Use Committee. *E/F; Mx1-cre* mice were injected with 1 mg pIpC (Sigma, Cat No. P0913) at 1 month of age to induce *Mx1* promoter. Blood was drawn from submandibular vein using 3 mm sterile animal lancets (MEDIpoint, Cat No. Goldenrod 3 mm) in microtainer tubes with EDTA (BD, Cat No. 365973) before pIpC injection and weekly afterwards. Blood smears were prepared from 2 μl of weekly blood draws onto single frosted micro slides (Corning 2948-75X25) and were stained using Diff-Quick staining kit (Dade Behring, Cat No. B4132-1A) per manufacturer's instruction. The images were captured by light microscopy on a Zeiss Imager Z.1 and pictures were taken using a connected PixeLINK camera (Nikon™) at an objective magnifications of 10x or 40x. WBC and RBC count is used to monitor disease state and progression in addition to enrichment for CD43^+^, CD71^+^, CD117^+^, and CD45^−^ cells in 50 μl of peripheral blood. For weekly RBC counts, 1 μl blood was first diluted 1:200 in 1x PBS and the number of RBC were then determined using hematocytometer. For weekly WBC counts, 2 μl of blood were first diluted 1:50 in 2% acetic acid to lyse RBC present in the blood and were then counted using hematocytometer. Once an increase in white blood cell (WBC) count to an average of 10,000 cells/μl and a 0.4–4% enrichment of CD43^+^, CD71^+^, CD117^+^, and CD45^−^ erythroblasts in the peripheral blood were observed, the mice were randomly assigned to treatment or control groups The animals were not enrolled if the disease was not induced during a maximum of four weeks follow up. For the short-term study in Figures 4 to 10, the animals received a total of 10 IP injections of YK-4-279 or control in 20 μl DMSO over a 15-day period. The first two injections were done at a dose of 200 mg/kg YK-4-279 or vehicle. For the remaining injections, the animals were administered with 150 mg/kg daily injections. The mice were then euthanized two weeks following enrollment to DMSO or YK-4-279 treatment. The long-term study in Figure 11 was carried out to assess the impact of YK-4-279 on survival. Here, the transgenic mice with EWS-FLI1 induced leukemia were randomly assigned to daily IP injections of 150 mg/kg YK-4-279 or vehicle five times a week for the first two weeks and were followed up for a maximum of three months. In both studies, only those animals that completed the entire two weeks long treatment regimen were included in the analysis.

### Immunohistochemistry

Paraffin sections were de-waxed and antigen retrieval was performed by heating in Citrate buffer (DAKO, S2369) in an autoclave for 10 min. Endogenous peroxidase was blocked by incubating the sections with 3% hydrogen peroxidase for 10 min. To prevent nonspecific stainings, several blocking steps were performed with avidin (Sigma-Aldrich, A9275), biotin (Sigma-Aldrich, B4501), superblock (ID labs, IDSTM003) and mouse block (ID labs, IDSTM003). The sections were incubated with specific antibodies against Gata1 (Santa Cruz, sc-265) in a 1:120 dilution, cleaved caspase 3 (Cell Signaling, 9661) in a 1:200 dilution, and Ki67 (Cell signaling, 9449) in a 1:1000 dilution at 4°C overnight. The next day, the sections were incubated with a biotinylated secondary antibody (ID labs, IDSTM003) and HRP (ID labs, IDSTM003) for 10 min. Specific signals were amplified using 3-Amino-9-Ethylcarbazole (ID laboratories, BP1108) under visual control followed by a counterstaining with haematoxylin (Merck, 1.092.491.000). The sections were mounted using Aquatex (Merck, 1.08562.0050). All antibodies were incubated overnight at 4°C and diluted in PBS+1% BSA. The images were taken with the Zeiss AxioImager Z1 microscope and were analyzed using HistoQuest software (TissueGnostics GmbH, Vienna, Austria, www.tissuegnostics.com). For the liver samples, only the infiltrating cells were counted and analyzed. Therefore, the settings of the cell recognition were made in a way that all the other cell types (e.g. hepatocytes) were not detected.

### Liver function test

Following a two weeks course treatment with either YK-4-279 or DMSO, the mice were euthanized and 1 ml of blood was collected by cardiac puncture and was transferred onto a tube that is not treated with anticoagulant to allow the blood to clot. The clot was removed by centrifugation at 200 g for 15 min at 4°C. The supernatant (serum) was flash frozen and stored in −80°C until use. Aspartate aminotransferase (AST) and alanine aminotransferase (ALT) levels in 50 μl serum samples from healthy *E/F; control* mice (*n* = 5) and leukemic *E/F; Mx1-cre* mice treated with DMSO (*n* = 12) or YK-4-279 (*n* = 12) was measured with a Reflotron Plus analyzer (Roche, Basel, Switzerland) per manufacturer's instruction.

### Statistical analysis

All Statistical analyses were performed using the GraphPad Prism version 4 (GraphPad software, La Jolla, CA, USA). Unpaired two-tailed *t*-test was utilized to assess whether the observed differences between different groups were statistically significant if the datasets being compared do not violet assumptions of the test. Welch's *t*-test test was utilized if the datasets being compared have unequal variance, which violates the assumption of a standard unpaired *t* test. Paired two-tailed *t*-test was used to determine whether the difference in the means between two time points of the same treatment group were statistically different. Kaplan-Meier survival curves were compared using logrank test.

## SUPPLEMENTARY FIGURES AND TABLE


