# Correction: YK-4-279 effectively antagonizes EWS-FLI1 induced leukemia in a transgenic mouse model

**DOI:** 10.18632/oncotarget.28524

**Published:** 2024-02-22

**Authors:** Tsion Zewdu Minas, Jenny Han, Tahereh Javaheri, Sung-Hyeok Hong, Michaela Schlederer, Yasemin Saygideğer-Kont, Haydar Çelik, Kristina M. Mueller, Idil Temel, Metin Özdemirli, Heinrich Kovar, Hayriye Verda Erkizan, Jeffrey Toretsky, Lukas Kenner, Richard Moriggl, Aykut Üren

**Affiliations:** ^1^Department of Oncology, Georgetown University Medical Center, Washington, DC, USA; ^2^Department of Pathology, Georgetown University Medical Center, Washington, DC, USA; ^3^Ludwig Boltzmann Institute for Cancer Research, Vienna, Austria; ^4^Clinical Institute of Pathology, Medical University of Vienna, Vienna, Austria; ^5^Unit of Pathology of Laboratory Animals, University of Veterinary Medicine, Vienna, Austria; ^6^Medical University of Vienna, Vienna, Austria; ^7^Children's Cancer Research Institute, St. Anna Kinderkrebsforschung, Vienna, Austria; ^8^Institute of Animal Breeding and Genetics, University of Veterinary Medicine, Vienna, Austria; ^9^Department of Pediatrics, Medical University of Vienna, Vienna, Austria; ^*^These authors have contributed equally to this work

**This article has been corrected:** In Figure 9, the low magnification liver image from E/F; Mx1-cre, treated with DMSO group (middle row) and the high magnification spleen image from the same group are accidental identical images. The low magnification liver image is correct. The corrected Figure 9, produced using the original data, is shown below. The authors declare that these corrections do not change the results or conclusions of this paper.

Original article: Oncotarget. 2015; 6:37678–37694. 37678-37694. https://doi.org/10.18632/oncotarget.5520

**Figure 9:**
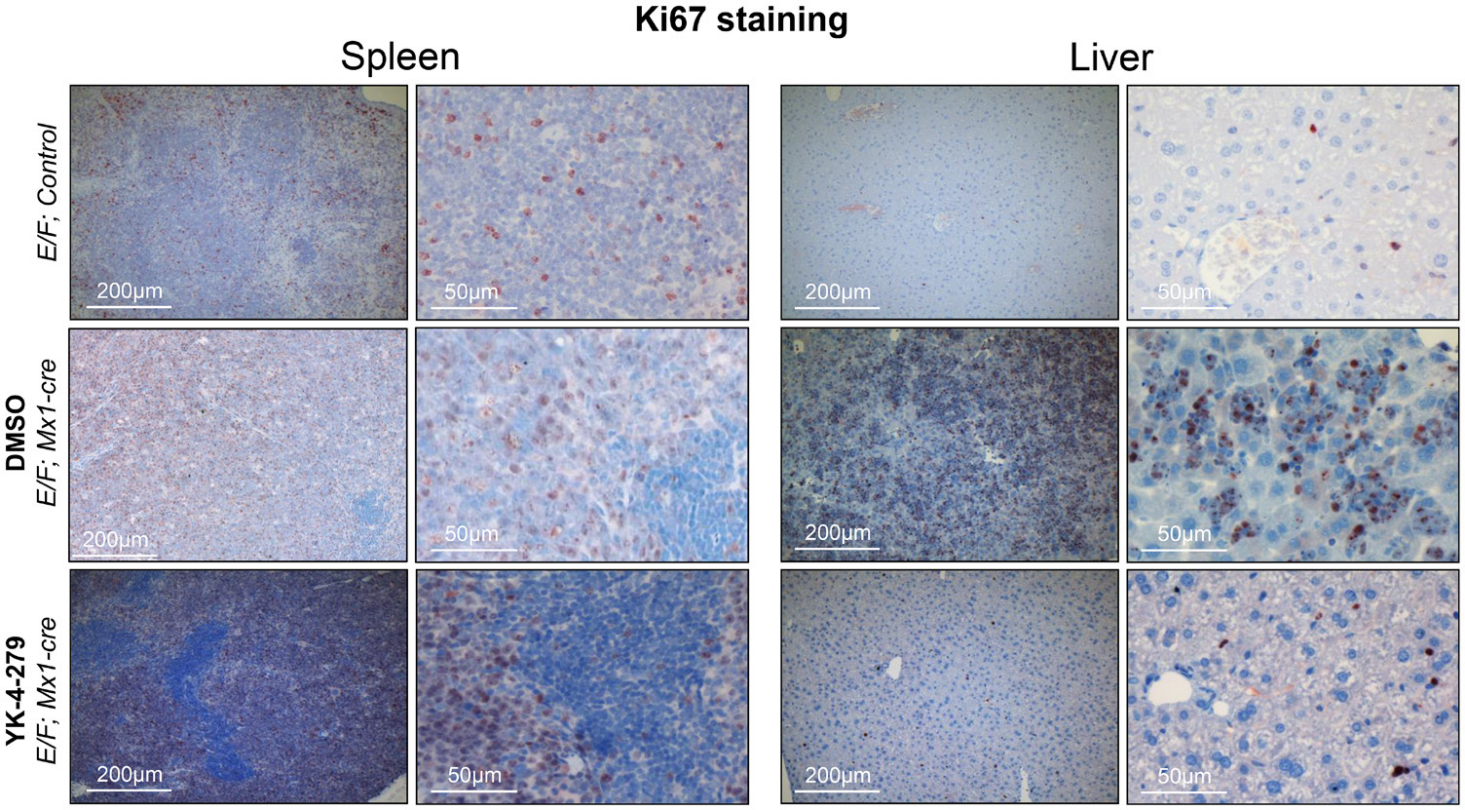
Decreased proliferation was observed in spleens and livers of YK-4-279 treated leukemic mice. *E/F; Mx1-cre* mice after two weeks treatment with vehicle (middle row) or YK-4-279 (bottom row) were euthanized and spleen and liver samples were processed for histo-pathology analysis. *E/F*; control mice that lack cre required for *EWS-FLI1* activation served as healthy controls (top row) which did not display any symptoms of disease or any peculiar apoptosis or proliferation. Ki67 was used as a marker of proliferation. Ki67 staining of spleen and liver tissues of leukemic *E/F;Mx1-cre* mice treated with YK-4-279 show a decreased proliferation compared to vehicle treated mice.

