# Supplementary material for: YK-4-279 effectively antagonizes EWS-FLI1 induced leukemia in a transgenic mouse model
Source: Oncotarget. 2015 Oct 8;6(35):37678–94. doi: 10.18632/oncotarget.5520 (PMC4741957; doi:10.18632/oncotarget.5520)
Supplement: Supplementary file 1 [file oncotarget-06-37678-s001.pdf]

SUPPLEMENTARY FIGURES AND TABLE

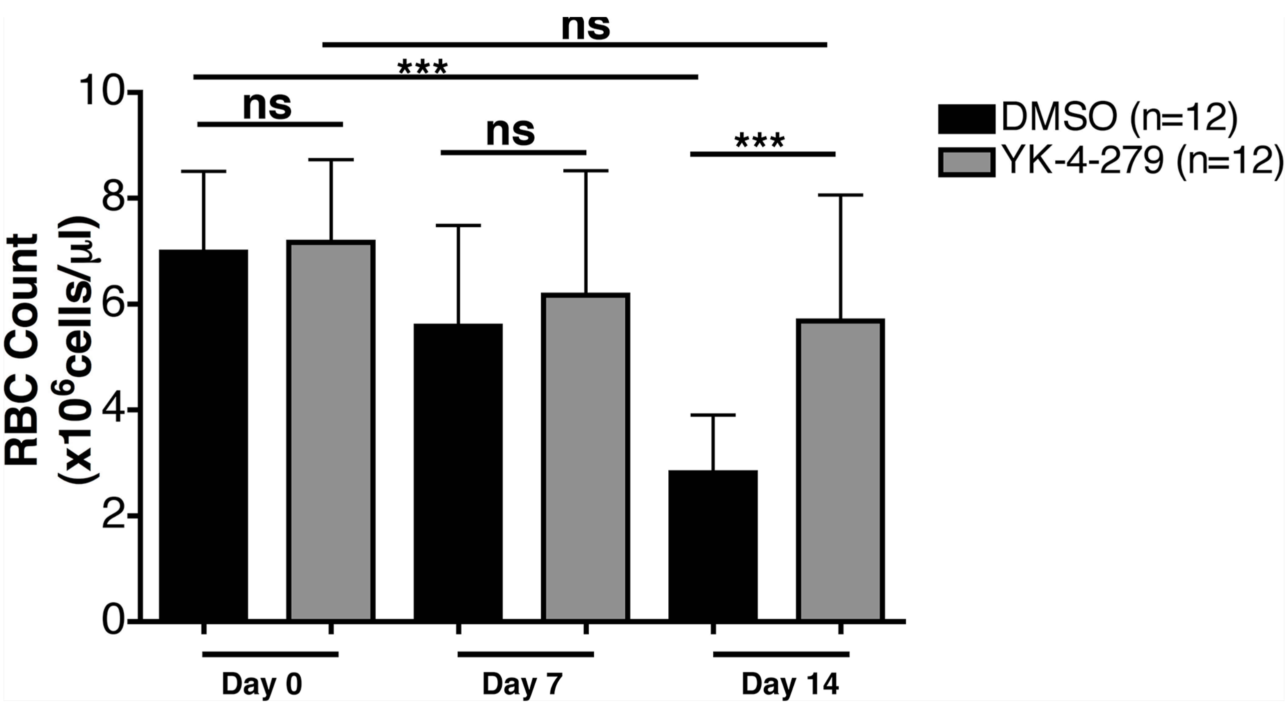

Supplementary Figure S1: YK-4-279 improved anemic state of mice with EWS-FLI1 induced leukemia. *E/F; Mx1-cre* mice with EWS-FLI1 induced leukemia present with sever anemia especially during the late stages of the disease. Red blood cell (RBC) count was utilized as an indicator of anemia status. RBC counts were determined from weekly blood draws at the day of treatment assignment (Day 0), and one week (Day 7) and two weeks (Day 14) following treatment. At the time of randomization to treatment, both DMSO and YK-4-279 group had similar RBC counts. The RBC count of DMSO group decreased significantly at the time of euthanasia (Day 14) while the RBC count of mice on YK-4-279 for two weeks remained unchanged. ***; $p < 0.0001$, ns; not-significant.

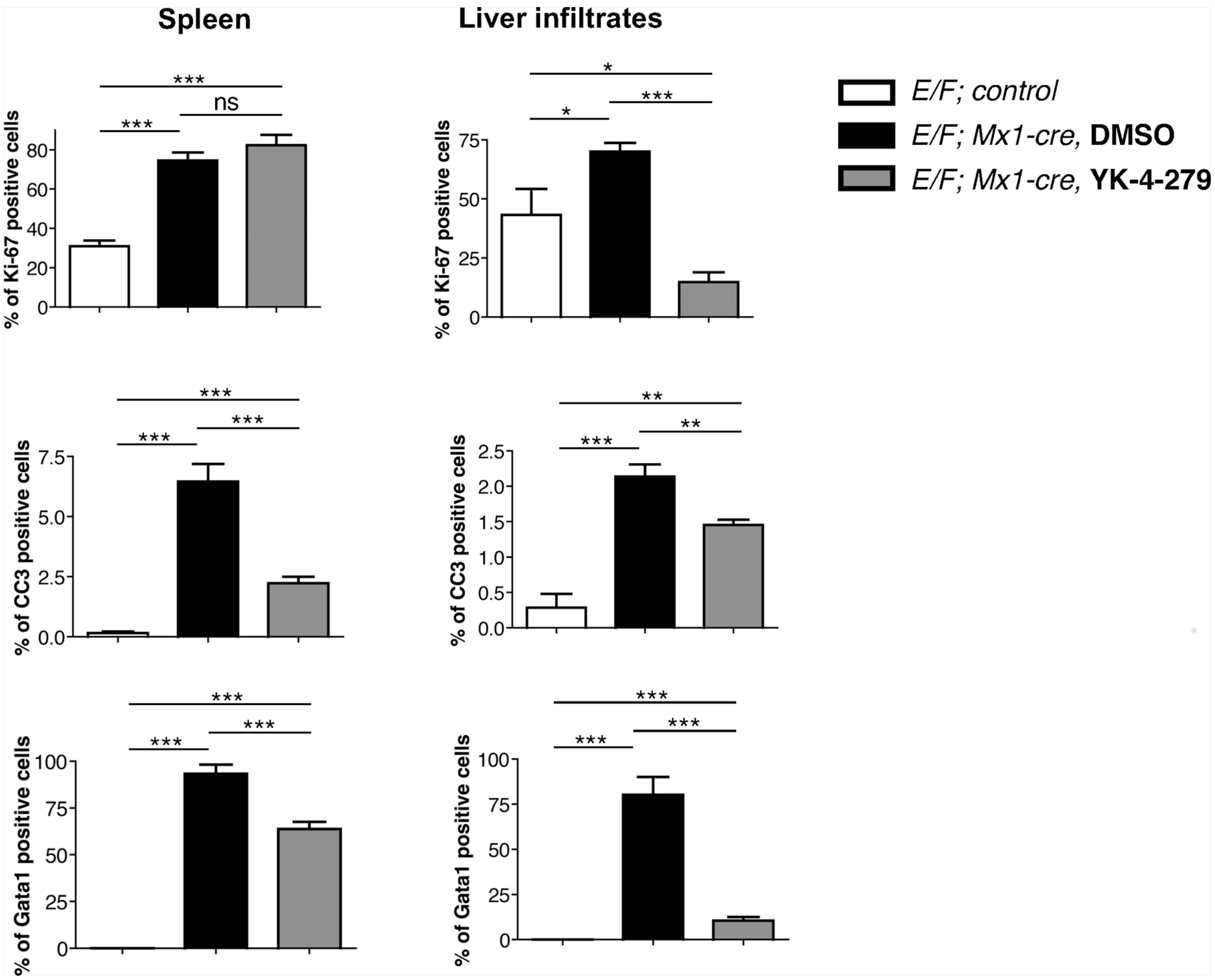

Supplementary Figure S2: Quantification of Ki-67, cleaved caspase 3, and Gata1-positive cells in the spleen and liver infiltrates of DMSO vs. YK-4-279 treated *E/F; Mx1-cre* leukemic mice as compared to healthy *E/F; control* mice. The images were taken with the Zeiss AxioImager Z1 microscope and were analyzed using HistoQuest software. For the liver samples, only the infiltrating cells were counted and analyzed. . *; $p < 0.05$, **; $p < 0.001$, ***; $p < 0.0001$, ns; not-significant.

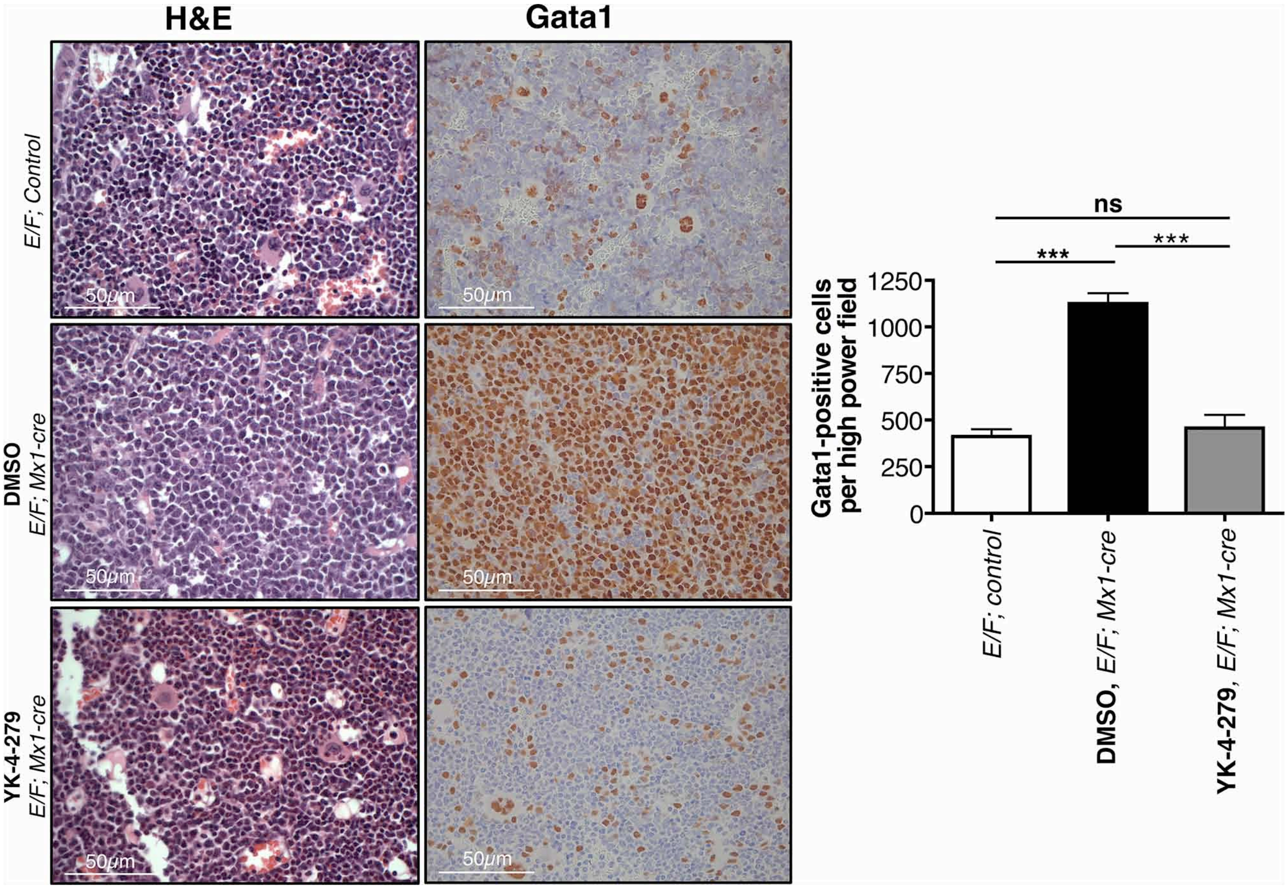

Supplementary Figure S3: Representative images of H&E and Gata1 stained bone marrow samples from study subjects. Bone marrows from three cohorts were analyzed: (1) Healthy *E/F; control* animals that did not have any transgene expression (Top), (2) *E/F; Mx1-cre* animals that developed rapid leukemia and received placebo injection (DMSO), (middle), and (3) *E/F; Mx1-cre* animals that received YK-4-279 for 2 weeks (bottom). *E/F; Mx1-cre* mice developed acute leukemia with numerous blasts. Normal maturing granulocytic cells, erythroid precursors and megakaryocytes were rare (H&E middle panel). *E/F; Mx1-cre* mice treated with YK-4-279 had hypercellular marrow with trilineage hematopoiesis including adequate megakaryocytes and rare blasts (H&E bottom panel) similar to control mice (H&E top panel). IHC staining of the same samples with a Gata1 antibody confirmed the therapeutic effect of YK-4-279. Number of Gata1 positive cells from each group were counted and presented in the bar graph. ***; $p < 0.0001$, ns; not-significant.

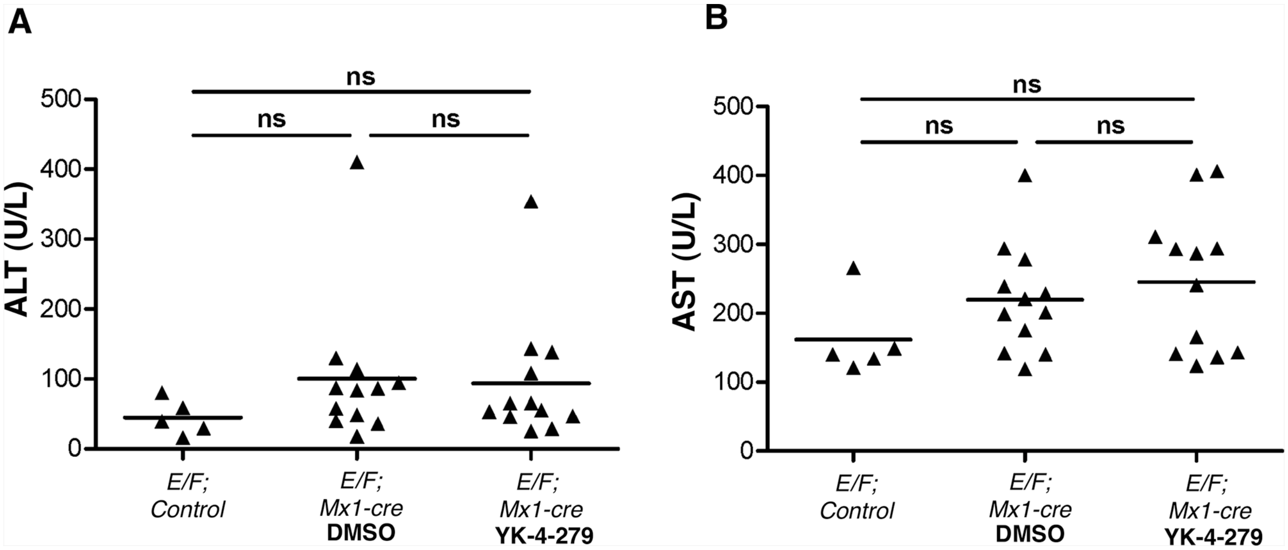

Supplementary Figure S4: A two-week course treatment with YK-4-279 did not affect liver function. Serum levels of liver enzymes **A.** alanine aminotransferase (ALT) and **B.** aspartate aminotransferase (AST) were measured to evaluate liver function of leukemic *E/F; Mx1-cre* mice following a two-week course treatment with either YK-4-279 or DMSO. *E/F; control* mice that lack *cre* required for EWS-FLI1 activation served as healthy controls. Severe erythroleukemia in these mice did not change overall liver function. YK-4-279 treatment did not impair liver function. ns; not-significant.

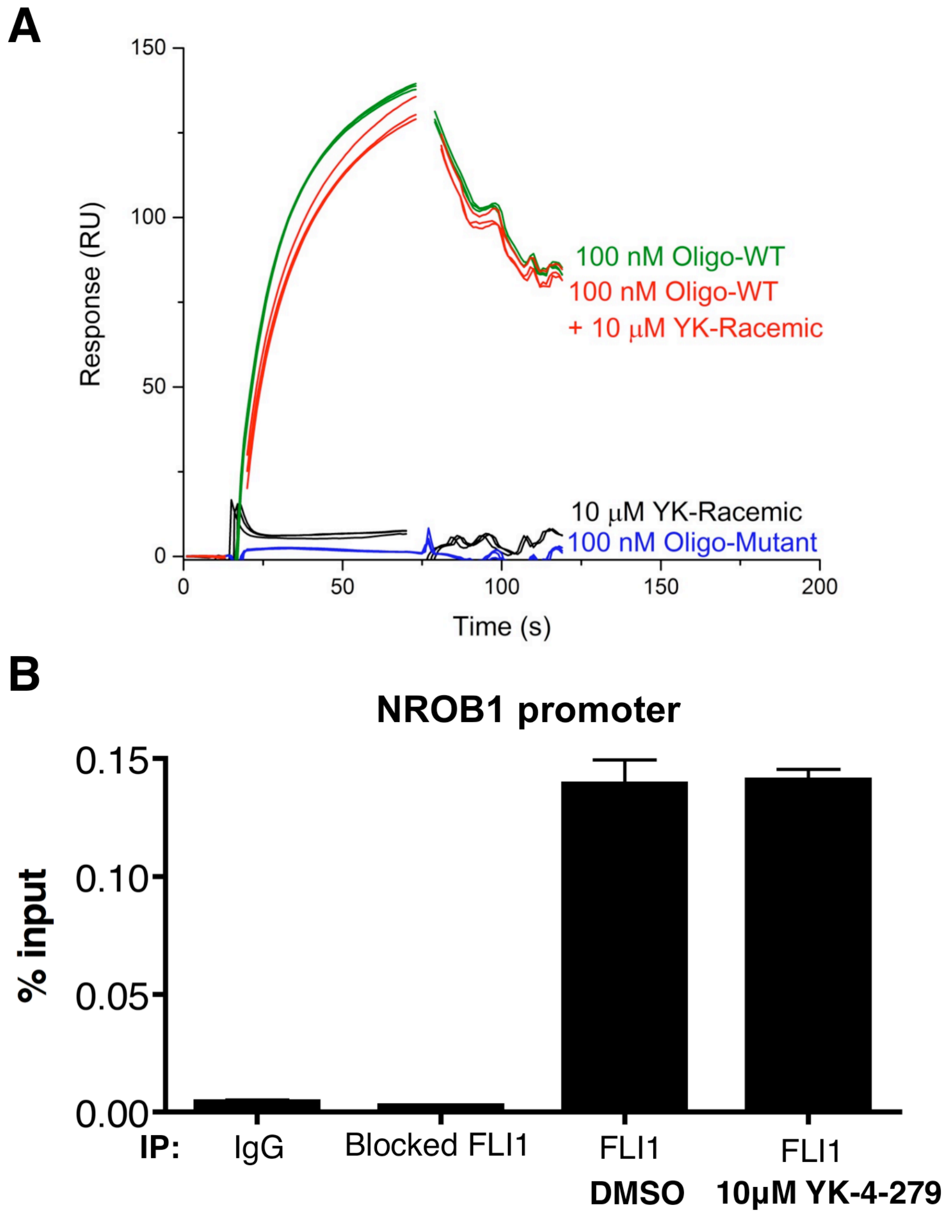

Supplementary Figure S5: YK-4-279 did not inhibit EWS-FLI1 DNA binding. A. Recombinant EWS-FLI1 protein was immobilized on the surface of a Biacore CM5 chip by amine coupling. Wild-type double-stranded oligonucleotides (*ATGTAGACC**GGAA**GTAACTA*) containing the consensus *ets* binding site "GGAA" were injected in 100nM triplicates over the surface of the chip in presence or absence of 10 µM YK-4-279. Binding of DNA to recombinant EWS-FLI1 was measured using Biacore T200 software. **B.** ChIP-qPCR assay was performed in TC71 cells that harbor endogenous EWS-FLI1. Cells were treated with 10 µM YK-4-279 or vehicle for 2 hrs. YK-4-279 did not inhibit binding of EWS -FLI1 to NR0B1 promoter.

Supplementary Table S1: List of qPCR primers

Gene symbol	Gene name	Forward primer	Reverse primer
Mest	Mesoderm specific transcript	ATCCCGGTGCTTCTTCTCA	AGGCAGCAAGCAGCAACT
Cpne7	Copine VII	CTAGTGGGCTGACTTGCCA	AATGGCCGTGTCATCTTCTT
c-Myc	Myelocytomatosis oncogene	CCCTATTTCATCTGCGACGAG	GAGAAGGACGTAGCGACCG
Car8	Carbonic anhydrase 8	ACCCCTCACTGCTGGATGTC	TGTCCATCGTTGGTGACTTCA
Gata1	GATA Binding Protein 1	GCCCAAGAAGCGAATGATTG	GTGGTCGTTTGACAGTTAGTGCAT
Gata2	GATA Binding Protein 2	GGCACGGGCCACTACCT	TGAGCGGCCGGTTCTG
EWS-FLI1		CAGCCTCCCACTAGTTACCC	GTTGAGGCCAGAATTCATG
